# Hydrous mantle plume promoted the generation of continental flood basalts in the Tarim large igneous province

**DOI:** 10.1038/s41598-024-60213-4

**Published:** 2024-04-25

**Authors:** Yao Bi, Huan Chen, Eero Hanski, Takeshi Kuritani, Hong-Xiang Wu, Feng-Qi Zhang, Jia Liu, Xiao-Yan Gu, Qun-Ke Xia

**Affiliations:** 1 CAS Key Laboratory of Crust-Mantle Materials and Environments, School of Earth and Space Science, University of Science and Technology of China, Hefei, 230026 China; 2 Institute of Marine Geology, College of Oceanography, Hohai University, Nanjing, 210098 China; 3 Key Laboratory of Geoscience Big Data and Deep Resource of Zhejiang Province, School of Earth Sciences, Zhejiang University, Hangzhou, 310027 China; 4 Oulu Mining School, University of Oulu, P.O. Box 3000, 90014 Oulu, Finland; 5 Graduate School of Science, Hokkaido University, Sapporo, 060-0810 Japan

**Keywords:** Geochemistry, Volcanology

## Abstract

Recent research on the water content of large igneous provinces (LIPs) has revealed that water has a significant impact on the formation of LIPs. However, most studies focus on the water content of mafic–ultramafic rocks, while relatively little attention has been paid to the water content of continental flood basalts (CFB), which form the major part of LIPs and are characterized by huge volumes (> 1 × 10^5^ km^3^) and short eruption times. Here, we determined water contents of clinopyroxene crystals from the Akesu diabase, which is co-genetic with flood basalts of the Tarim LIP in China. Based on these measurements, we obtained a water content of higher than 1.23 ± 0.49 wt.% for the parental magma to the Tarim CFB and a minimum water content of 1230 ± 490 ppm for the mantle source, thus indicating the presence of a hydrous mantle plume. Combined with previous studies, our results suggest that water plays a key role in the formation of the Tarim LIP. Additionally, the whole-rock compositions of the Akesu diabase indicate a contribution of pyroxenite in the mantle source. This is consistent with a model, in which water was brought into the Tarim mantle plume by a subducted oceanic plate that entered the deep mantle.

## Introduction

Flood basalts are the main constituents of large igneous provinces (LIPs). Their huge magma volumes (> 1 × 10^5^ km^3^) but short eruption times (usually < 1–3 Ma)^1,2^ imply unique geodynamic processes, and therefore the origin of LIPs has been a hot topic in Earth sciences^2^.

The view according to which the formation of LIPs is related to mantle plumes originated from the core-mantle boundary is widely accepted^2–4^. Previous studies have found that these mantle plumes generally have an abnormally high temperature^4,5^, indicating that temperature is one of the important factors to the formation of LIPs. In addition, experimental petrology has demonstrated that water lowers the solidus temperature of rocks^6–8^ and reduces the viscosity of magmas^9,10^, which may contribute to the formation of LIPs.

In recent years, studies of the water content in LIP magmas have gradually increased^11–23^. Based on the small-scale mafic–ultramafic rocks (e.g., picrites, meimechites, komatiites and mafic intrusive rocks), they have constrained the water content in the LIPs, including the Emeishan LIP (2.71–3.73 wt.%)^11,12^, the Siberian LIP (0.25–3.88 wt.%)^13,14^, the Karoo LIP (1–2 wt.%)^15^, the Caribbean LIP (0.25–0.70 wt.%)^16–18^, and the Tarim LIP (4.82 ± 1.00 wt.%)^19^. In addition, Stefano et al.^20^, Cabato et al.^21^ and Choudhary et al.^22^ measured the water content of melt inclusions in Snake River Plain basalts (SRPB), Columbia River basalts (CRB) and Deccan Trap basalts, respectively, and found that these basalts likely have a high water contents as well (up to 2.4 wt.%, 3.3 wt.% and 2.05 wt.%, respectively). Based on these studies above, Liu et al.^11^ estimated that the water content of the mantle source in the LIPs ranges from 900 to > 6000 ppm, and the H_2_O/Ce ratios range from 160– 400 to > 2000, which are significantly higher than those of MORBs (50–250 ppm, 150–210)^24,25^ or most OIBs (300–1000 ppm, < 250)^26,27^. Thus, they proposed that water is an important factor in the formation of LIPs^11–23^. However, most of the water content data are from small-scale mafic–ultramafic rocks, and the data from continental flood basalts (CFB) are still relatively scares. Besides, CFB often undergo intense crystal fractionation and the host minerals of melt inclusions are not the earliest-crystallized^20–22^ phases in previous studies, making it difficult to directly use the measured water contents to obtain information on the mantle sources. Hence, the water contents in CFB and the effects of water on CFB are not yet well understood.

**Figure 1:**
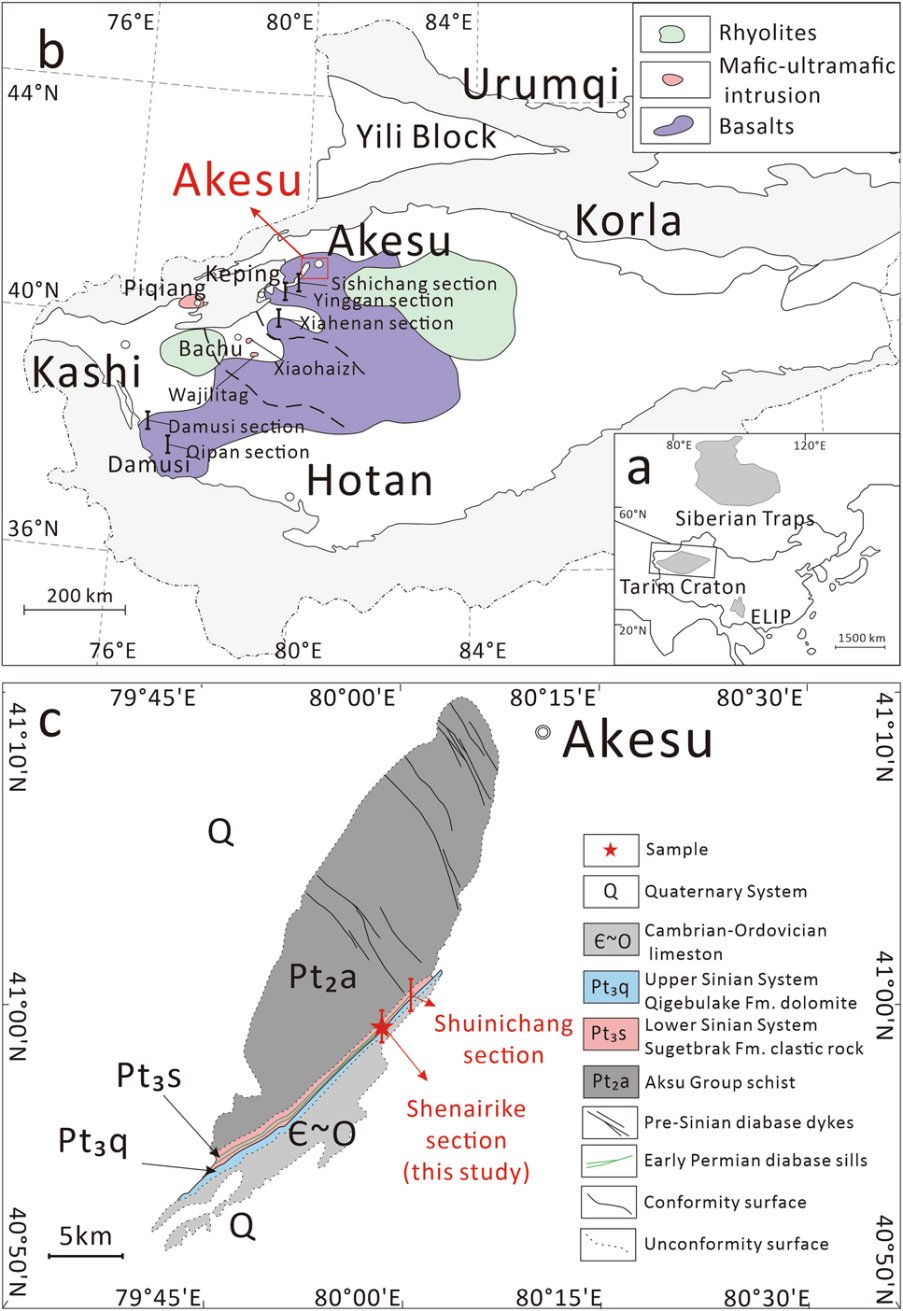
(**a**) Map showing the location of the Tarim LIP (Modified after Zhou et al.^32^). (**b**) Simplified geologic map of the Tarim LIP (modified after Xu et al.^29^) showing the distribution of CFB and mafic–ultramafic intrusive rocks. (**c**) Geological sketch map of the Akesu section (modified after Turner^66^).

The Tarim LIP is a typical LIP of the Phanerozoic Eon. It is located within the Tarim Basin in northwest China with a residual area of approximately 250,000 km^2^ and a maximum thickness of up to 800 m^28,29^ (Fig. 1a, b). Except for the small-volume kimberlites emplaced at ~ 300 Ma^30^, the Tarim LIP can be divided into two stages: the main stage (290–288 Ma) produced large-scale CFB^28,29^ and the later stage (284–278 Ma) small-scale mafic–ultramafic intrusive rocks^28,29^. Liu et al.^11^ calculated a high mantle potential temperature for picrites, indicating that the formation of the Tarim LIP is related to a mantle plume. Xia et al.^19^ found that late-stage intrusive rocks have a high water content. In addition, based on numerical simulation, Liu and Leng^31^ also proposed that the formation of the Tarim LIP is related to a water-rich mantle plume. However, the water content of the CFB in the Tarim LIP is still unclear, and therefore this work focuses on determining water contents of the Tarim CFB.

The Tarim CFB generally have low MgO contents^28,29,32,33^ (Supplementary Fig. S1), indicating that they have undergone intense fractional crystallization, which means that their water contents do not represent those of the primary melts. Recently, Wu et al.^34^ reported the presence of sheet-like mafic intrusive rocks (diabases) with a thickness of 6–8 m in the Akesu area (Fig. 1c). These rocks intruded into the Lower Cambrian Sugetbark Formation, forming a discordant body. Geochronological studies indicate that the formation age of these rocks is between 292 and 290 Ma^34,35^, which is consistent with the age of the Tarim CFB rather than that of the mafic–ultramafic intrusive rocks belonging to the later magmatic stage^28,29,32,33,36^. In addition, the trace element characteristics and Sr–Nd isotope compositions of the Akesu diabase are similar to those of the Tarim CFB^32^. These results confirm that the Akesu diabase is co-genetic with the Tarim CFB. It is noteworthy that the MgO content of the Akesu diabase (up to 8.71 wt.%)^35^ is generally higher than that of other Tarim CFB (2.05–6.99 wt.%)^28,29,32,33^ (Supplementary Fig. S1), indicating that the diabase is less evolved and can better reflect the information on the mantle source of the Tarim CFB.

In this study, six samples of the Akesu diabase with large clinopyroxene phenocrysts (18SK3-2, 18SK3-4, 13-AKS-19a, 14-AKS-19a, 11-AKS-19a and 11-AKS-19b) from previous studies^34,35^ were selected (Supplementary Fig. S1 and S2). The diabase generally exhibits a low porosity, appearing as dense blocks with localized spherical weathering. Petrographic microscope shows that these diabases are ophitic texture, but commonly contain large clinopyroxene phenocrysts (Supplementary Fig. S3). The matrix is primarily composed of plagioclase and pyroxene microlites, and volcanic glass^34,35^. Water contents of clinopyroxene phenocrysts were measured using unpolarized infrared spectra and major and trace element contents of clinopyroxene phenocrysts of these samples were determined with an electron microprobe and LA-ICP-MS instrument, respectively. Based on mass balance calculations, the water content and H_2_O/Ce ratio in the mantle source were estimated and the impact of water on the formation of LIPs is discussed.

## Results

### Water content and chemical composition of clinopyroxene in the Akesu diabase

Major and trace element compositions of clinopyroxene phenocrysts are shown in Supplementary Table S1. The Mg# (Mg# = molar ratio of 100 × Mg^2+^/(Mg^2+^ + Fe^2+^)) of clinopyroxene phenocrysts ranges from 65.6 to 74.0, which are too low to be in equilibrium with the melt of the Akesu diabase (Supplementary Fig. S4). Based on the partition coefficients of trace elements between clinopyroxene and melt^37^, the trace element composition of the equilibrated melt was calculated. As shown in Supplementary Fig. S5, the trace elements pattern of the equilibrated melt is consistent with that of Akesu diabase, which features indicate that the clinopyroxene phenocrysts were crystallized from the magma of their host rocks^37^.

**Figure 2:**
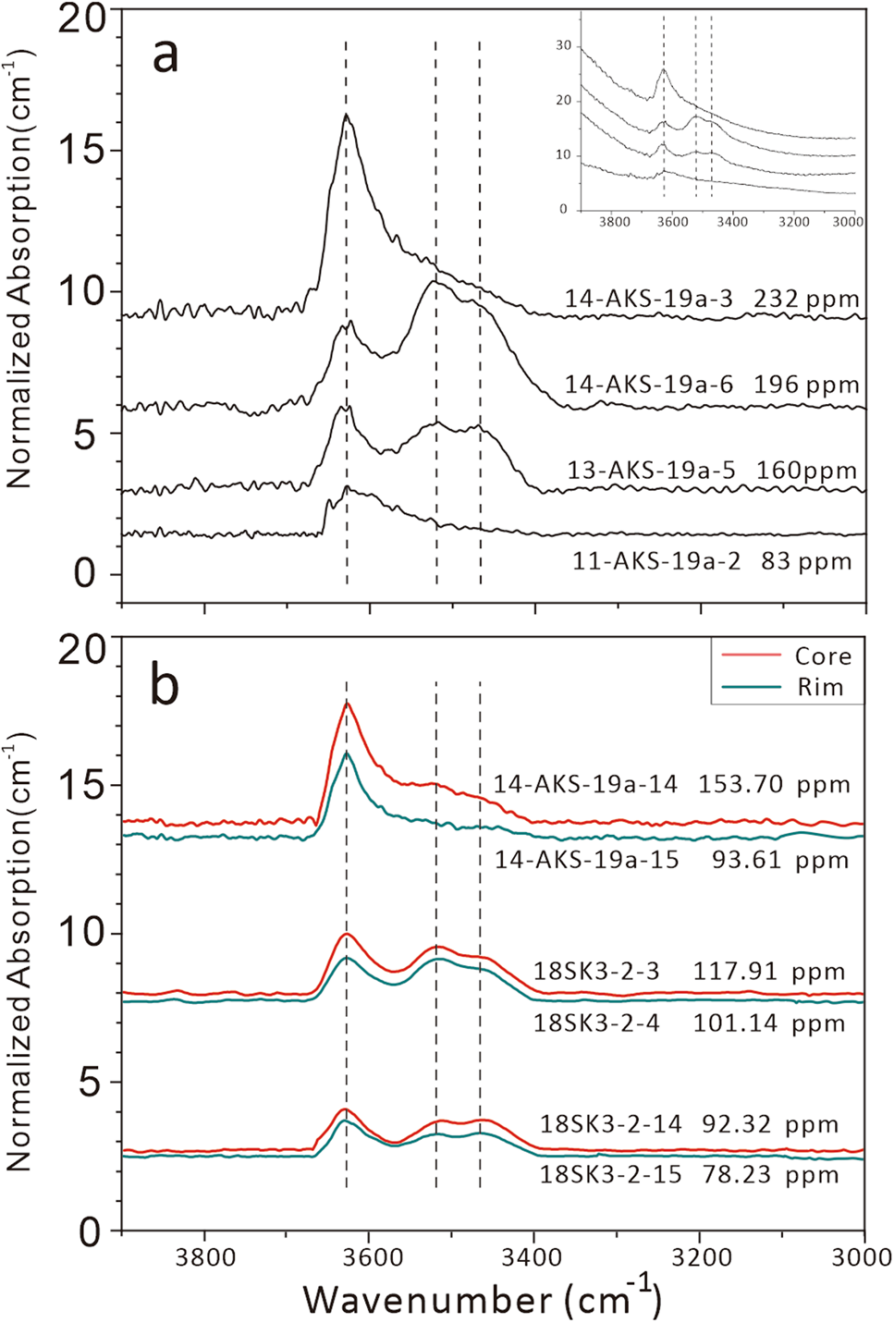
(**a**) Representative IR spectra of OH in clinopyroxene phenocrysts from the Akesu diabase. (**b**) Representative IR of profiles for clinopyroxene phenocrysts from the Akesu diabase. The vertical axis is normalized absorbance to 1 cm and the baseline has been corrected. The dashed lines mark the position of individual OH bands. The insert shows spectra without baseline correction.

In the OH stretching vibration region (3000–3800 cm^−1^), the clinopyroxene in the Akesu diabase exhibits three main bands at ~ 3640 cm^−1^, ~ 3530 cm^−1^, and ~ 3460 cm^−1^ (Fig. 2a), of which the band at 3640 cm^−1^ is strongest. These results are consistent with data from clinopyroxene phenocrysts in basalts reported previously^38,39^. The maximum linear absorbance band for all clinopyroxenes is less than 0.15, meaning that the unpolarized infrared spectra measurement is reliable with an error < 30%^40^. Based on the Beer-Lambert law, the calculated water content of clinopyroxene grains in the Akesu diabase ranges from 20.7 to 253.0 ppm (see Supplementary Table S1 and Supplementary Fig. S6. The detailed calculation process is reported in the Supplementary Text).

The water content of the melt in equilibrium with the clinopyroxene was calculated using mass balance and the coefficient ${\text{D}}_{{\text{H}}_{2}{\text{O}}}^{\text{cpx-melt}}$ using the following equation ^41^:

1 $${\text{D}}_{{\text{H}}_{2}{\text{O}}}^{\text{cpx-melt}}= \text{ E} \text{xp (-} \, \text{4.2} \, \text{+} \, \text{6.5} \times { \, {\text{X}}}_{{\text{Al}}^{\text{IV}}}^{\text{cpx}} -{ \, {\text{X}}}_{\text{Ca}}^{\text{cpx}})$$

where ${\text{X}}_{{\text{Al}}^{\text{IV}}}^{\text{cpx}}$ and ${\text{X}}_{\text{Ca}}^{\text{cpx}}$ are the stoichiometric numbers of four-coordinated Al and Ca in the chemical equation of clinopyroxene phenocrysts (under 6 oxygen atoms). The calculated water content of the melt equilibrated with clinopyroxene phenocrysts ranges from 0.17 to 1.87 wt.%.

For clinopyroxene phenocrysts with the maximum linear absorbance band less than 0.3, the maximum deviation in measuring of a single clinopyroxene phenocryst is less than 19%^40,42^. Taking into account an error of 10% for the I, the error of calculated the water content of a single clinopyroxene phenocryst by unpolarized light is < 30%^,^^40,42,43^. Considering the typical uncertainty of ${\text{D}}_{{\text{H}}_{2}{\text{O}}}^{\text{cpx-melt}}$ ^41^(~ 10%), the total uncertainty of the calculated water content in the melt is less than 40%^40,42,43^.

## Discussion

### The effects of alteration on the Akesu diabase

In surface environments, rocks may undergo alteration, leading to the changes in the water content. So, it is necessary to assess the effects of alteration, on the Akesu diabase. before evaluating its water content.

The Akesu diabase exhibits high levels of loss on ignition (LOI 3.2–5.3 wt.%)^35^, indicating a relatively strong effect of alteration. In particular, some samples with high LOI values have anomalous contents of fluid-mobile elements (e.g., K, Rb; Supplementary Fig. S7 and S8), indicating that their chemical composition has been strongly affected by alteration. However, the K and Rb contents of the samples selected for this study are relatively consistently low (Supplementary Fig. S7), suggesting that the effect of alteration on this samples is limited. Furthermore, the infrared spectroscopy is highly sensitive to the hydroxy, can effectively identify different types of structural water and molecular water, allowing for the check whether minerals have undergone alteration or not. Therefore, we were able to select fresh clinopyroxene grains for analysis of the water content and chemical composition to avoid the influence of alteration.

### Crustal contamination and fractional crystallization

During magma evolution, the water content of melt may be influenced by crustal contamination and fractional crystallization. Compared to the mantle, the continental crust is generally enriched in U and Pb, but depleted in Ce and Nb^44^. Thus, when magma is contaminated by the material from continental crust, a positive correlation in the ratios of Nb/U to Ce/Pb or Nb/U to Nb can be identified. However, for samples with lesser influence from alteration, the lack of a relationship between Nb/U and Ce/Pb or Nb/U and Nb (Supplementary Fig. S9) suggests that crustal contamination did not significantly affect the Akesu diabase.

While the MgO content of the Akesu diabase is higher compared to other Tarim CFB, it is still relatively low in general, which means that the Akesu diabase has undergone intense fractional crystallization. As shown in Supplementary Fig. S10, the Akesu diabase has a low concentration of Ni and Cr, and the Ni and Cr display a positive correlation with MgO, respectively. These phenomenon in Supplementary Fig. S10 suggests that Akesu diabase primarily underwent fractional crystallization of the olivine and clinopyroxene, as indicated by the enrichment of Ni in olivine and Cr in clinopyroxene. Moreover, if fractional crystallization of plagioclase takes place, the magma would exhibit negative anomalies in Eu due to the compatibility of Eu with plagioclase. However, the absence of an obviously Eu anomaly in the rare earth element pattern diagram of the Akesu diabase (Supplementary Fig. S8) indicates that the crystal differentiation of plagioclase is insignificant.

### Estimation of the melt water content of the Akesu diabase

The calculated trace element composition of the melt in equilibrium with the clinopyroxene phenocrysts displays a similar pattern to the Akesu diabase (Supplementary Fig. S5c, d), indicating that these clinopyroxene phenocrysts crystallized from the Akesu diabase magma. This means that clinopyroxene grains can be utilized to infer the water content of the magma.

During crystallization, H is an incompatible element and consequently the water content of the residual melt would increase. To eliminate the effects of crystallization, the earliest-crystallized phenocrysts should be used to calculate the water content of the primitive melt. However, all clinopyroxene phenocrysts in the diabase have relatively low Mg# (65.6–74.0) as a result of strong crystal fractionation and hence the water content in the melt was modified by crystal fractionation.

As mentioned earlier, the water content of the Akesu diabase is influenced by fractional crystallization of olivine and clinopyroxene. In order to constrain the water content of the primary melt, we incrementally added olivine and clinopyroxene to the melts in certain proportions, with 1% incremental steps. The calculation was stopped when the melt Mg# reaches 72. The simulation process involved the design of three different scenarios. In the first scenario, olivine and clinopyroxene were introduced into the melt in a 1:1 ratio. In the second scenario, the ratio was adjusted to 2:1, with a higher proportion of olivine to clinopyroxene. The third scenario involved solely adding olivine to the melt. For the calculation of H_2_O content in melt, we employed specific H partition coefficients and Fe–Mg exchange coefficients. The H partition coefficient for olivine was set at 0.0002^45^, while the Kd for olivine was determined as 0.30^46^. Additionally, we utilized a H partition coefficient of 0.015 (calculated from clinopyroxene in Akesu diabase) and Kd of 0.33^46^ for clinopyroxene.

The simulation results, as depicted in Supplementary Fig. S11, indicate an overestimation of the water content in the melt by 52.0% (1:1), 43.2% (2:1), and 30.8% (only olivine) in the three scenarios, respectively.

Considering that olivine and clinopyroxene are the primary crystallized phases during magma evolution in Akesu diabase, we utilized a 1:1 ratio of olivine to clinopyroxene to estimate the minimum water content. Then the water content of the equilibrated melts was determined to be 0.11 to 1.23 wt.%.

On the other hand, H profile analyses across clinopyroxene phenocrysts show that the water content at the rim is generally lower than that in the core (Fig. 2b). This indicates outward diffusion of H in clinopyroxene phenocrysts.

**Figure 3:**
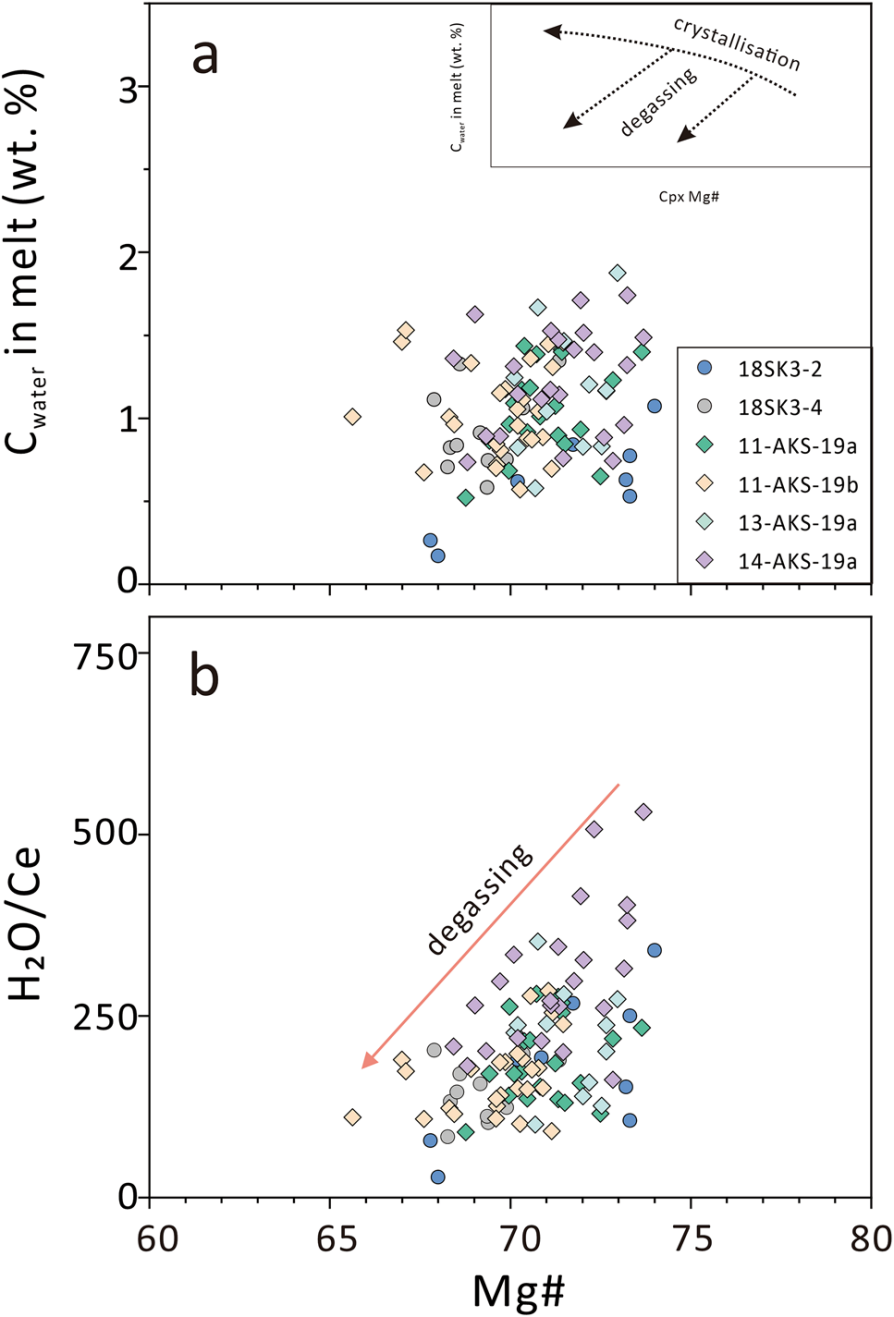
(**a**) Water contents of equilibrated melts and (**b**) calculated H_2_O/Ce ratios of equilibrated melts versus Mg# values of clinopyroxene phenocrysts in the Akesu diabase. The inserted schematic plot illustrates the variation in H_2_O-Mg# trends during the processes of crystallization and degassing (modified after Wade et al.^47^). The partition coefficient for water between clinopyroxene and equilibrated melt is from Eq. 10 in O’Leary et al.^41^. The partition coefficient for Ce between clinopyroxene and equilibrated melt is from Eqs. 304, 306, and 307 in Bédard^48^. The arrows show the effects of degassing^47^.

As shown in the Fig. 3a, calculated water content of the equilibrated melt varies widely and demonstrate a slight decrease with the evolution of magma, (especially for 18SK3-2). In theory, due to the strong incompatibility of hydrogen (H), the water content in magma tends to continuously increase during the process of fractional crystallization^47^. However, this is different from features observed in Fig. 3, suggesting the degassing of magma. Furthermore, the Akesu diabase exhibits a significant variation in water content, as depicted in Fig. 3a. This wide range of water content suggests the occurrence of degassing processes or the lack of homogenization in clinopyroxene phenocrysts.

In order to minimize the influence of whole-rock composition, the H_2_O/Ce ratio of the equilibrated melt was calculated based on the major and trace element compositions of clinopyroxene phenocrysts. The partition coefficient of Ce was estimated by averaging values obtained from Eqs. 304, 306, and 307 in Bédard^48^, while the partition coefficient of H_2_O was determined using Eq. 10 in O'Leary et al.^41^. Figure 3b demonstrates a notable positive correlation between the H_2_O/Ce ratio and the Mg# of the samples. Previous studies have shown that H_2_O and Ce exhibit similar incompatibility in basaltic melts^25,26^. This suggests that there should be minimal changes in the H_2_O/Ce ratio during partial melting and fractional crystallization. When comparing the variations of 1/Ce and H_2_O/Ce, it is evident that the trend in 1/Ce shows much smaller changes compared to the trend in H_2_O/Ce (Supplementary Fig. S12). This indicates that the observed trend in Fig. 3b can be attributed to degassing rather than an enrichment of Ce in the melt.

According to the research conducted by Patkó et al.^49^, clinopyroxene found in peridotite samples from the northern Pannonian Basin exhibited infrared spectra where the dominant OH absorption band at 3630 cm^−1^ was not prominent (the ratio of absorption intensities at approximately 3630 cm^−1^ and ~ 3525 cm^−1^ wavenumbers was less than 1.2). They concluded that these clinopyroxene phenocrysts experienced equilibration under conditions of lower water activity, resulting in the loss of water content. However, in the case of Akesu diabase, the clinopyroxene phenocrysts displayed consistent features with other basalts^40,42,43^, showing a dominant OH absorption band at 3630 cm^−1^. This discrepancy indicates that the clinopyroxene phenocrysts in Akesu diabase did not undergo a similar process as observed in the peridotite samples from the northern Pannonian Basin^49^.

Consequently, we can conclude that the water content of the primitive melt is underestimated. Considering both the impact of crystal fractionation and water diffusion, it is likely that the water content of the primitive melt in the Tarim CFB was higher than 1.23 ± 0.49 wt.%.

The estimated water content of the Tarim CFB is similar to those of the other CFB (SRPB, 0.2–3.3 wt.%^20^; CRB, 0.6–4.24 wt.%^21^; Deccan flood basalt, 0.94–2.05 wt.%^22^ and Emeishan flood basalt, 2.71 ± 0.95 wt.%)^23^ and the small-scale mafic–ultramafic rocks in LIPs (0.25–4.83 wt.%)^11–18^ and is significantly higher than that of OIBs (0.1–0.3 wt.%)^50^ and MORBs (0.3–1.0 wt.%)^10^.

### High water content and H_2_O/Ce ratio in the mantle source of the Tarim CFB

The water content in the mantle source of the Tarim CFB can be calculated using batch melting and fractional melting models and the following equations:

Batch melting

2 $${\text{C}}_{{0} \, }= \text{ } {\text{C}}_{\text{L}}\text{[} \, {\text{F}} \, \text{+} \, \left({1} \, - \, {\text{F}}\right) \, \times \, \text{D]}$$

Fractional melting

3 $${\text{C}}_{0}\text{ = }{\text{C}}_{\text{L}} \frac{\text{F}}{\text{[1} \, - \, \text{(1} \, - \, {\text{F}}{)}^{\frac{1}{{\text{D}}}}\text{]}}$$

where ${\text{C}}_{0}$ is the water content in the mantle source, ${\text{C}}_{\text{L}}$ is the water content of the melt, F is the degree of partial melting, and D is the bulk partition coefficient of water between melt and the mantle minerals (D = 0.0088–0.013)^7^.

**Figure 4:**
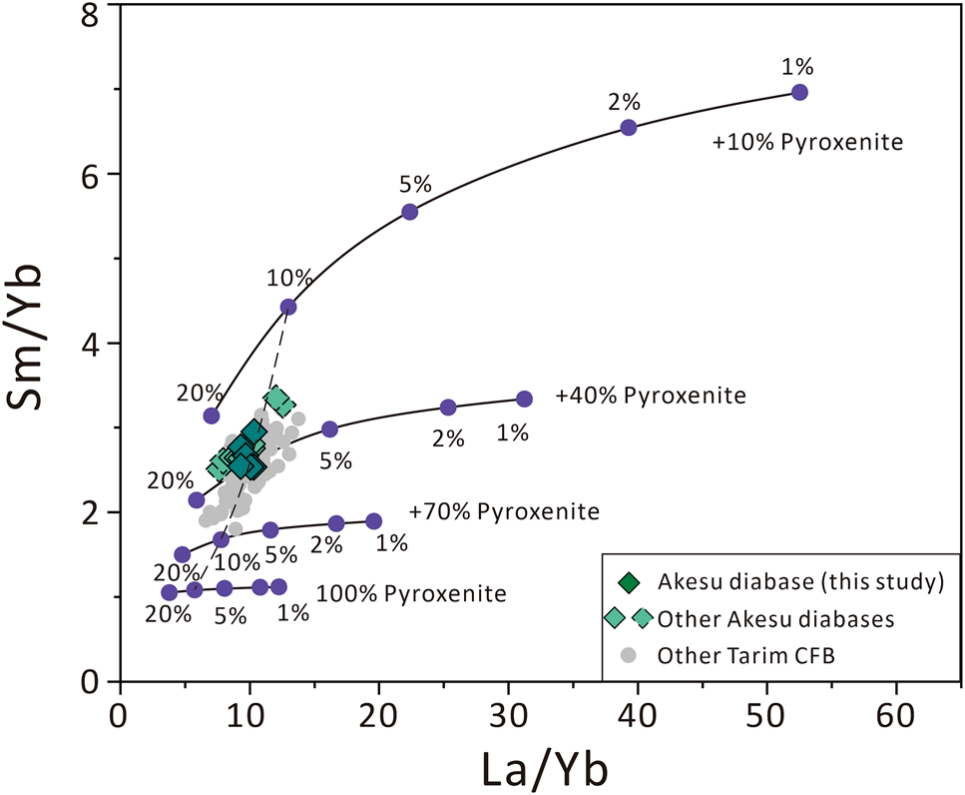
Sm/Yb versus La/Yb diagram showing partial melting degrees for the Akesu diabase magma and other Tarim CFB (modified after Cheng et al.^54^). The mantle source is assumed to be composed of carbonated peridotite and pyroxenite, with peridotite containing 55% olivine, 13% orthopyroxene, 15% clinopyroxene 15% garnet and 2% calcite. Pyroxenite is composed of clinopyroxene. The dashed line represents mixing curves between melts derived from peridotite and pyroxenite. The dashed line represents mixing curves between melts derived from peridotite and pyroxenite. The data symbols with dashed borders represent samples that have been strongly affected by alteration. The chemical compositions of the Akesu diabase samples selected for water content measurements and the other Akesu diabase samples are all from Cheng et al.^35^. The contents of La, Sm and Yb are from McDonough and Sun^67^, and mineral/melt partition coefficients for La, Sm and Yb are from Rollison^68^. The data of other Tarim CFB are from Keping basalts^28,29,32,33,54^.

Based on the fractionation of rare earth elements, the degree of partial melting for the Akesu diabase magma was estimated to be approximately 10% (Fig. 4). Our calculation indicates that the minimum water content in the mantle source of the Tarim CFB is 1330 ± 530 ppm for the batch melting model and 1230 ± 490 for the fractional melting model (Supplementary Table S2).

**Figure 5:**
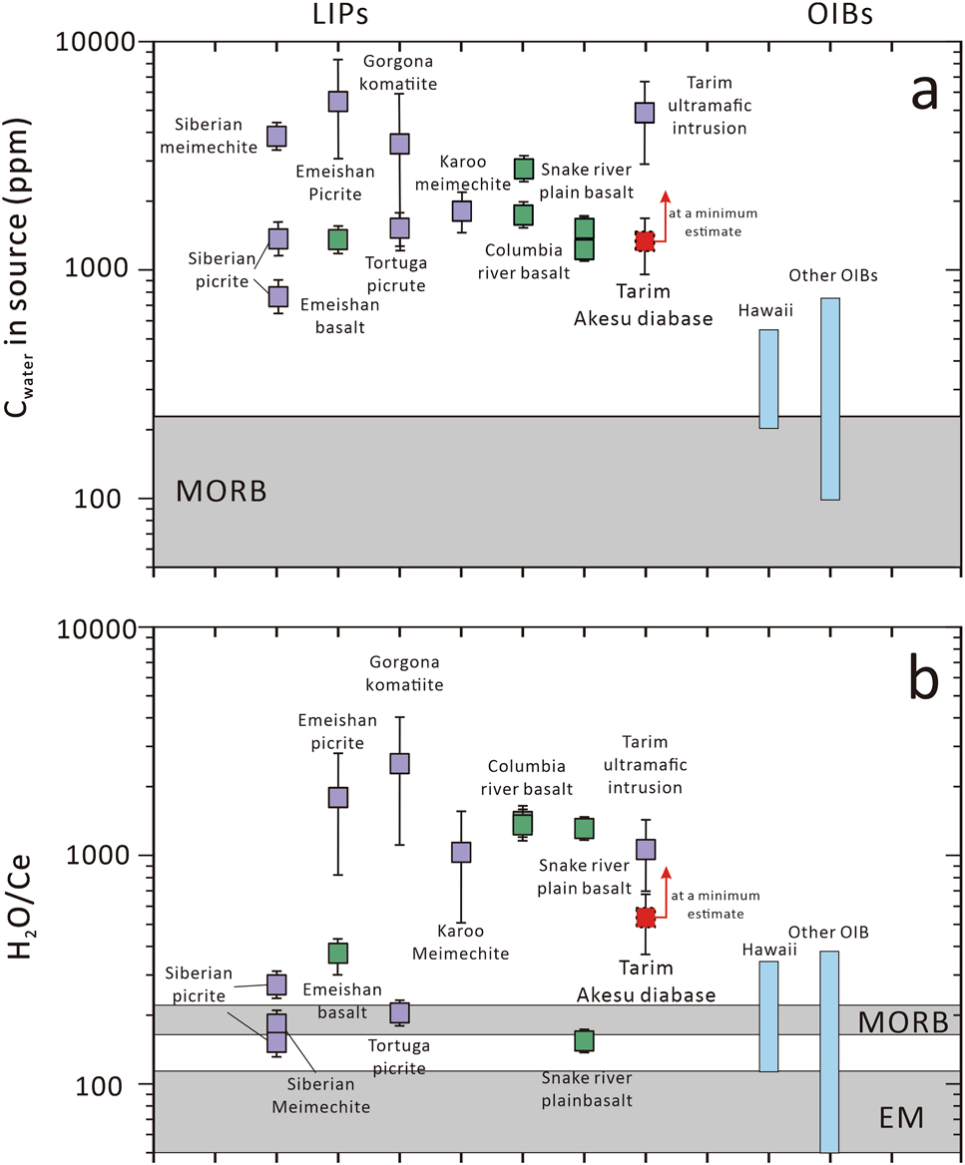
Comparison of (**a**) the water contents and (**b**) H_2_O/Ce ratios in the mantle source of LIPs and OIBs (modified after Liu et al.^11^). The purple boxes represent small-scale mafic–ultramafic rocks in LIPs, and the green boxes represent CFB. The ranges of Hawaii and other OIBs are from Bizimis and Peslier^26^. Data of LIPs are from the Siberian Trap^13,14^, Emeishan LIP^11,12,23^, Caribbean LIP^16–18^, Karoo LIP^15^, Columbia River basalts (CRB)^21^, Snake River Plain basalts (SRPB)^20^ and Tarim LIP^19,52^.

As shown in Fig. 5a, the mantle source of other CFB, including SRPB (940–2257 ppm)^20^, CRB (2257–3869 ppm)^21^, and Emeishan basalt (1357–1579 ppm)^23^, exhibit similar water contents to that of the Akesu diabase. In the case of small-scale mafic–ultramafic rocks found in LIPs, the water content in their mantle source ranges from 750 to higher than 6000 ppm^11–19^ (Fig. 5a). It is evident that the mantle source of both CFB and mafic–ultramafic rocks in LIPs contain much more water than those of MORBs (50–250 ppm)^24,25^ or OIB (300–1000 ppm)^26,27^.

As mentioned earlier, the H_2_O/Ce ratio in magma remains stable during processes of partial melting and fractional crystallization^51^. This indicates that H_2_O/Ce in the melt can provide information on the ratio in the mantle source. Our calculations reveal that the H_2_O/Ce ratios of the melt ranges from 30 to 500 (as illustrated in Fig. 3b). However, considering the effects of hydrogen diffusion, the H_2_O/Ce ratio of the mantle source likely exceeds 500.

According to Fig. 5b, the H_2_O/Ce ratios of the Akesu diabase are similar to those in other LIPs (150 to  > 2000)^11–21,23^, and the majority of LIPs have higher H_2_O/Ce ratios than MORBs (150–210)^24,25^. The results indicate that the water content in the mantle source of the Tarim CFB is higher than 1230 ± 490 ppm and H_2_O/Ce ratio exceeds 500. When combined with previous data from late-stage mafic intrusive rocks (4820 ppm, 1043)^19,52^, it can be concluded that the water content and H_2_O/Ce ratio in the mantle source of the Tarim LIP, falling within the range of LIPs (Fig. 5), are significantly higher than those of MORBs and OIBs^24,25^.

The presence of water in the mantle source can lower the solidus of rocks, cause melting to occur at greater depths, and increase the volume of the generated magma^6–8^. This likely contributed to the formation of the large-scale CFB observed in the Tarim LIP. Additionally, water can decrease the density and viscosity of magma, which in turn can increase the rate of magma ascent, promoting the formation and upward movement of mantle plumes. These observations suggest that water plays a key role in the formation of the Tarim LIP.

### The origin of the water in the Tarim CFB

**Figure 6:**
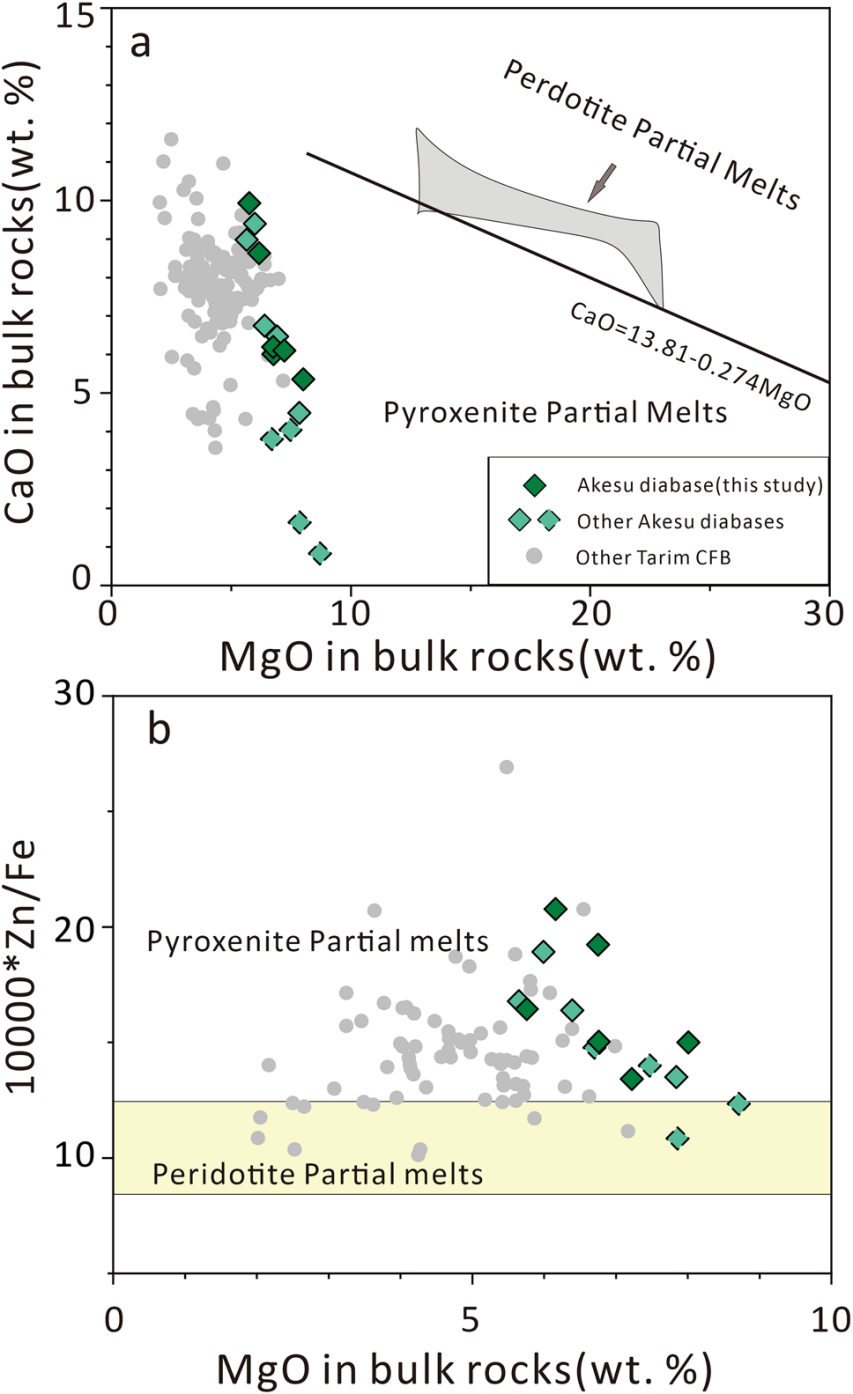
(**a**) CaO and (**b**) 10,000*Zn/Fe versus MgO diagram for bulk rock samples from the Akesu diabase and other Tarim CFB. The line separating pyroxenite and peridotite melts in (**a**) is from Herzberg and Asimow^69^. The ranges of 10,000*Zn/Fe in peridotite and pyroxenite melts in (**b**) are from Le Roux et al.^70^. The data sources for the Akesu diabase and other Tarim CFB are same as in Fig. 4.

As a part of the Tarim CFB, the Akesu diabase exhibits lower CaO and higher Zn/Fe ratios than melts derived from peridotite (Fig. 6), which suggests the existence of pyroxenite components in the mantle source. Previous studies^53–55^ have shown that the Tarim continental flood basalts have lower Mg and higher Zn isotope ratios compared to the primitive mantle, suggesting the involvement of recycled oceanic plate in the mantle source. During the subduction of plates, basaltic oceanic plates, in the form of eclogite, can be subjected to partial melting and react with the peridotite mantle. This process can result in the formation of pyroxenite^56^. Taken together, these findings suggest that the mantle source of the Tarim CFB involved a subducted oceanic plate component.

Subducted oceanic plates play a crucial role in transporting water into the Earth's interior. During subduction of oceanic plates, fluids are released and metasomatize the mantle wedge, leading to the generation of water-rich island arc basalts (e.g., Ryukyu)^57^. Due to the depletion of high field strength elements (e.g., Nb, Ta) in the released fluids, island arc basalts typically exhibit significant negative anomalies in Nb and Ta^57^, However, this is inconsistent with the trace element features of the Akesu diabase (Supplementary Fig. S8), which indicates that the water in the mantle source of Akesu diabase does not originate from the metasomatized mantle wedge.

As the oceanic plates continue to subduct, released fluids can further metasomatize the continental lithospheric mantle, the released fluids can further metasomatize the lithospheric mantle, forming a hydrated metasomatized lithospheric mantle similar to the Mesozoic North China Craton (NCC)^40,58^. According to previous studies, the Tarim CFB can be divided into Group-1 and Group-2 based on the Sr–Nd isotope characteristics^59^. Compared to Group-2, Group-1 displays an enriched Sr–Nd isotope composition, which is supposed to originate from the lithospheric mantle. As shown in Supplementary Fig. S13, The Sr–Nd isotope composition of the Akesu diabase falls within the range of Group-1. Additionally, using machine learning, Zhang et al.^60^ suggested that the mantle source of the Tarim LIP is hydrous, and the fluid activity in the Tarim LIP decreases from northeast to southwest, which may be correlated with fluids released from earlier southward subduction of oceanic plate. This propose that the Akesu diabase may originate from the metasomatized lithospheric mantle.

The analogy can be drawn to the Mesozoic NCC, which serves as a prime example of a lithospheric mantle that has undergone metasomatism and is enriched in water^40,58^. The NCC experienced the continuous subduction of the Pacific Plate during the Mesozoic. As a result, fluids derived from the subducting Pacific Plate infiltrated the lithospheric mantle, leading to the development of a mantle source with significantly high water content. Notable examples of water-enriched basalts in the eastern region of the NCC include Feixian (> 1000 ppm in mantle source)^40^, Yixian (> 1500 ppm in mantle source)^58^, and Sihetun (> 1200 ppm in mantle source)^58^. These Mesozoic basalts consistently exhibit trace element characteristics of Nb–Ta depletion, as illustrated in Supplementary Fig. S8.

While the Akesu diabase also displays weak negative anomalies in Nb–Ta, they are distinctly different from those observed in the Mesozoic NCC basalts (as shown in Supplementary Fig. S8). This disparity suggests that the water found in the mantle source of the Akesu diabase may not primarily originate from a metasomatized lithospheric mantle in a manner similar to the Mesozoic NCC. Furthermore, a high water content (> 1000 ppm) in the lithospheric mantle can potentially induce destabilization and thinning processes^40^. However, the Tarim Craton has been recognized as a region characterized by geological stability throughout its history^61,62^.

It is worth noting that the oceanic plates can further subduct into the deep mantle. Seismological observations have shown that oceanic plates can subduct into the lower mantle^63^, and experimental petrology has demonstrated the existence of hydrous mineral phases in subducted oceanic plates that can remain stable under the high pressure and temperature conditions of the lower mantle^64^. This suggests that the water from subducted plates can be transported into the deep mantle. When subducted material enters into the mantle plume, it can give rise to the formation of water-rich basalts, which do not exhibit negative anomalies in Nb and Ta, such as Emeishan LIP^11,12,23^ and Columbia River Basalts^21^. Moreover, Zhang et al.^60^ suggested the mantle plume in the Tarim LIP might be hydrous. Additionally, based on numerical simulations, Liu and Leng^31^ concluded that the mantle plume of Tarim LIP exhibit characteristics of a water-rich composition. All of these studies indicate that the water in the mantle source of Tarim may originate from a mantle plume and be associated with subducted oceanic plates.

On the other hand, the finding of hydrous ringwoodite in ultra-deep diamonds suggests that the mantle transition zone could serve as a significant reservoir for storing water in the Earth's interior^65^. As the mantle plume passes through the mantle transition zone, melting can occur and transport water from the mantle transition zone into the mantle plume. This could also be a contributing factor to the high water content observed in the Tarim mantle plume.

## Conclusions

Considering the effects of fractional crystallization and degassing, it was estimated that the water content of the primitive Tarim CFB magma is higher than 1.23 ± 0.49 wt.%, and the minimum water content and H_2_O/Ce ratio in the mantle source is 1230 ± 490 ppm and 500, respectively, indicating the potential presence of a hydrous mantle plume under the Tarim Basin.

Because of the existence of a recycled oceanic plate component in the mantle source of the Tarim CFB, the water in the Tarim mantle plume may have originated from the hydrated subducted oceanic plates or the mantle transition zone.

## Methods

### Analysis of the water content in clinopyroxene phenocrysts

The analysis of the water content in clinopyroxene phenocrysts was carried out in the FTIR (Fourier-transform infrared spectroscopy) laboratory at the School of Earth Sciences, Zhejiang University. The water content of clinopyroxene crystals was analyzed using a Nicolet iS50 FTIR spectrometer equipped with a Continuμm microscope under unpolarized light, with a liquid nitrogen-cooled MCT-A detector. Dry air was used to sweep the infrared equipment and optical path throughout the process. During measurement, the background and number of scans were set to 128 times, the resolution was 4 cm^−1^, and the wavelength range was 1000–4500 cm^−1^. The measurements were focused on the center of crystals avoiding cracks or inclusions. The spot radius varied from 30 to 50 μm depending on the sample. For large crystals, multi-point measurements of the core–edge were performed. Finally, the thickness of each infrared analysis area was measured using a Digimatic Indicator ID-F150.

### In-situ analysis of major elements in clinopyroxene phenocrysts

The in-situ major element analysis of clinopyroxene phenocrysts was conducted using a Shimadzu EPMA 1720 electron probe at the Electron Probe Microanalysis Laboratory of Zhejiang University's School of Earth Sciences.

The measurement conditions were as follows: acceleration voltage of 15 kV, current of 20 nA, beam spot diameter of 1 μm, and counting time of 30 s for each element (10 s for characteristic bands and 10 s for front and rear background each). Natural minerals and artificially synthesized oxides were used as standard samples, and the ZAF correction process was used to obtain the major elements content of clinopyroxene phenocrysts. For the obtained data, see Supplementary Table S1.

### In-situ analysis of trace elements in clinopyroxene phenocrysts

In-situ trace element analyses of clinopyroxene phenocrysts were conducted in the LA-ICP-MS (laser ablation inductively coupled plasma mass spectrometry) laboratory of the School of Earth Sciences, Zhejiang University. The parameters of Analyte HE laser ablation system were as follows: laser wavelength of 193 nm, maximum energy density of 45 J/cm^2^, frequency of 6 Hz, pulse energy of 4 J/cm^2^, and ablation beam spot diameter of 60 μm. During measurement, the blank background acquisition time was 10 s, the sample ablation time was 40 s, and there was a 30 s purge between two ablations to clean the injection system. The total time for single-point analysis was 65 s. High-purity helium gas was used as the carrier gas, and the flow rates of this gas in the sample chamber and channel were set to 0.6 and 0.3 L/min, respectively. The mass spectrometer model was iCAPRQ (Thermofisher), and the parameters were set to: 14 L/min plasma cooling gas flow, 0.8 L/min auxiliary gas flow, 0.9 L/min sample gas flow, 1500 W radio frequency power, and a dwell time of 10 ms for each element.

Artificially synthesized glass NIST SRM 610 was used as the external standard, and the Ca content of clinopyroxene analyzed by electron probe was used as the internal standard for result calibration. The standard samples used during analysis were: NIST SRM 610 (artificially synthesized glass), SRM 612 (artificially synthesized glass), BCR-2G (natural glass) and BHVO-2G (natural glass from Hawaii volcanoes). The analysis accuracy of all trace elements was better than 20%. The results are shown in Supplementary Table S1.

### Supplementary Information

Supplementary Information.

Supplementary Tables.

## Data Availability

All the data used in this study are listed in Supplementary Data.
