# Supplementary material for: Hydrous mantle plume promoted the generation of continental flood basalts in the Tarim large igneous province
Source: Sci Rep. 2024 Apr 25;14:9514. doi: 10.1038/s41598-024-60213-4 (PMC11045731; doi:10.1038/s41598-024-60213-4)
Supplement: Supplementary file 1 — Supplementary Information. [file 41598_2024_60213_MOESM1_ESM.docx]

**Hydrous mantle plume promoted the generation of continental flood basalts in the Tarim Large Igneous Province.**

Yao Bi^1^, Huan Chen^2,3*^, Eero Hanski^4^, Takeshi Kuritani^5^, Hong-Xiang Wu^3^, Feng-Qi Zhang^3^, Jia Liu^3^, Xiao-Yan Gu^3^, Qun-Ke Xia^3^

^1^CAS Key Laboratory of Crust-Mantle Materials and Environments, School of Earth and Space Science, University of Science and Technology of China, Hefei, 230026 China

^2^Institute of Marine Geology, College of Oceanography, Hohai University, Nanjing, 210098 China

^3^Key Laboratory of Geoscience Big Data and Deep Resource of Zhejiang Province, School of Earth Sciences, Zhejiang University, Hangzhou, 310027 China

^4^Oulu Mining School, University of Oulu, P.O. Box 3000, 90014 Oulu, Finland

^5^Graduate School of Science, Hokkaido University, Sapporo 060-0810, Japan

*Correspondence author (email: huanchen@hhu.edu.cn)

**Supplementary Texts**

**Geological Background**

The Tarim Early Permian large igneous province (LIP) is located in northwest China (Fig. 1), with a residual area of approximately 250,000 km^2^ and a maximum thickness of up to 800 m^1,2^. The Tarim LIP is mainly composed of continental flood basalt (CFB), mafic-ultramafic intrusive rocks, felsic rocks and small volume of kimberlite^1-3^.

The Tarim CFB is the main constituents of the Tarim LIP and widely distributed in the Tarim Basin (Fig. 1b). The Tarim CFB mainly outcrops in the Keping area (e.g., the Yingan section, the Sishichang section, the Xiahenan section)^4-9^, and also in the southwestern Tarim basin, such as the Damusi section and the Qipan section^10,11^. Yu *et al*.^5^ conducted a systematic geochronological study on the Yingan section using zircon U-Pb dating and determined that the CFB erupted as early as 290 Ma (289.5±2.0 Ma) and the latest eruption occurred at 288 Ma (288.9±3.4 Ma). In addition, Yang *et al*.^10^ and Wei *et al*.^6^ conducted ^40^Ar-^39^Ar isotopic dating on the basalt from the Qipan and Yingan section, respectively. The obtained formation age is 290.1±3.5 Ma, 287.3±4.0 Ma, and 287.9±3.1 Ma, respectively, which is consistent with previous work^5^. Recently, Zhong *et al*.^12^ constrained the formation time of the Tarim CFB using the CA-TIMS zircon U-Pb dating on volcanic tuffs, which is between 289.77 ± 0.95 Ma and 284.27 ± 0.39 Ma. Therefore, the formation age of the Tarim CFB is about 290~288 Ma^5,6,10,12^.

Compared to the CFB, the mafic-ultramafic intrusive rocks in Tarim LIP are found on a smaller scale, and distributed in the Bachu, Wajilitage and Piqiang areas^6-9,13^. Yu *et al*.^4^ conducted a geochronological study on quartz syenite porphyries which crosscut the mafic intrusive rocks, and determined the formation age of 278.4±2.2 Ma. On the other hand, the Wajilitage layered intrusions yielded the SHRIMP U–Pb zircon age of 284.3 ± 2.2 Ma^13^. These studies constrained that the formation age of mafic-ultramafic intrusive rocks is between 284 and 278 Ma^4,13^, which is later than the Tarim CFB.

Recently, Wu *et al*.^14^ reported the presence of sheet-like mafic intrusive rocks (diabase) in the Akesu area, with a thickness of 6-8 m (Fig. 1c)^14^. The diabase sheet is conformably induced into the clastic rocks in the Sugetbrak Formation, which belongs to the Lower Cambrian. Wu *et al*.^14^ and Cheng *et al*.^15^ obtained formation ages of 291.3 ± 3.1 Ma, 292.1 ± 5.4 Ma, and 290 ± 6 Ma, respectively. This indicates that the formation age of the Akesu diabase is about 292~290 Ma. It’s consistent with the Tarim CFBs, but significantly earlier than that of the mafic-ultramafic intrusive rocks in Tarim LIP. Moreover, Cheng *et al.*^15^ conducted geochemical analyses on the Akesu diabase and found that it is not only consistent with Tarim CFB in terms of trace element compositions but also in Sr-Nd isotopes. Coupled with geochronological works, it’s suggested that Akesu diabase is co-genetic with the Tarim CFB.

From the Akesu diabase samples collected at the Shenairike section in previous studies^14,15^, we selected 6 relatively fresh samples. Based on the microscopic observation of rock sections, these Akesu diabases are ophitic texture, but commonly contain large clinopyroxene phenocrysts (with a diameter of up to 500μm, Supplementary Fig. S3). In the matrix, the clinopyroxene appears granular, while the plagioclase appears slender and tabular.

**Calculation of the water content in single clinopyroxene phenocryst**

The water content of clinopyroxene phenocrysts can be calculated using the transformed Beer-Lambert Law:

$\text{C}\text{ }\text{=}\text{ }\frac{\text{A}}{\text{I}\text{ }\text{×}\text{ }\text{t}}$ (1)

Where C represents the water content of clinopyroxene phenocrysts (ppm), A is the total absorption intensity of OH bands (cm^-1^), I is the characteristic absorption coefficient (7.09×10^-6^ ppm^-1^cm^-2^, from Bell and Rossman^16^), and t is the thickness of the phenocrysts (cm). Using unpolarized light to measure clinopyroxene phenocrysts, the A can be calculated as the integral area of absorption bands from phenocrysts multiplied by three^17-20^.

For clinopyroxene phenocrysts with the maximum linear absorbance band less than 0.15, the maximum deviation in measuring of a single clinopyroxene phenocryst is less than 19%^16-20^. Taking into account an error of 10% in the I, the error in measuring the water content of a single clinopyroxene phenocryst by unpolarized light is < 30%^19,20^.

The calculated water content of clinopyroxene phenocrysts from the Akesu diabase ranges from 20.7 to 253.0 ppm (Supplementary Table S1).

**Supplementary Figures**


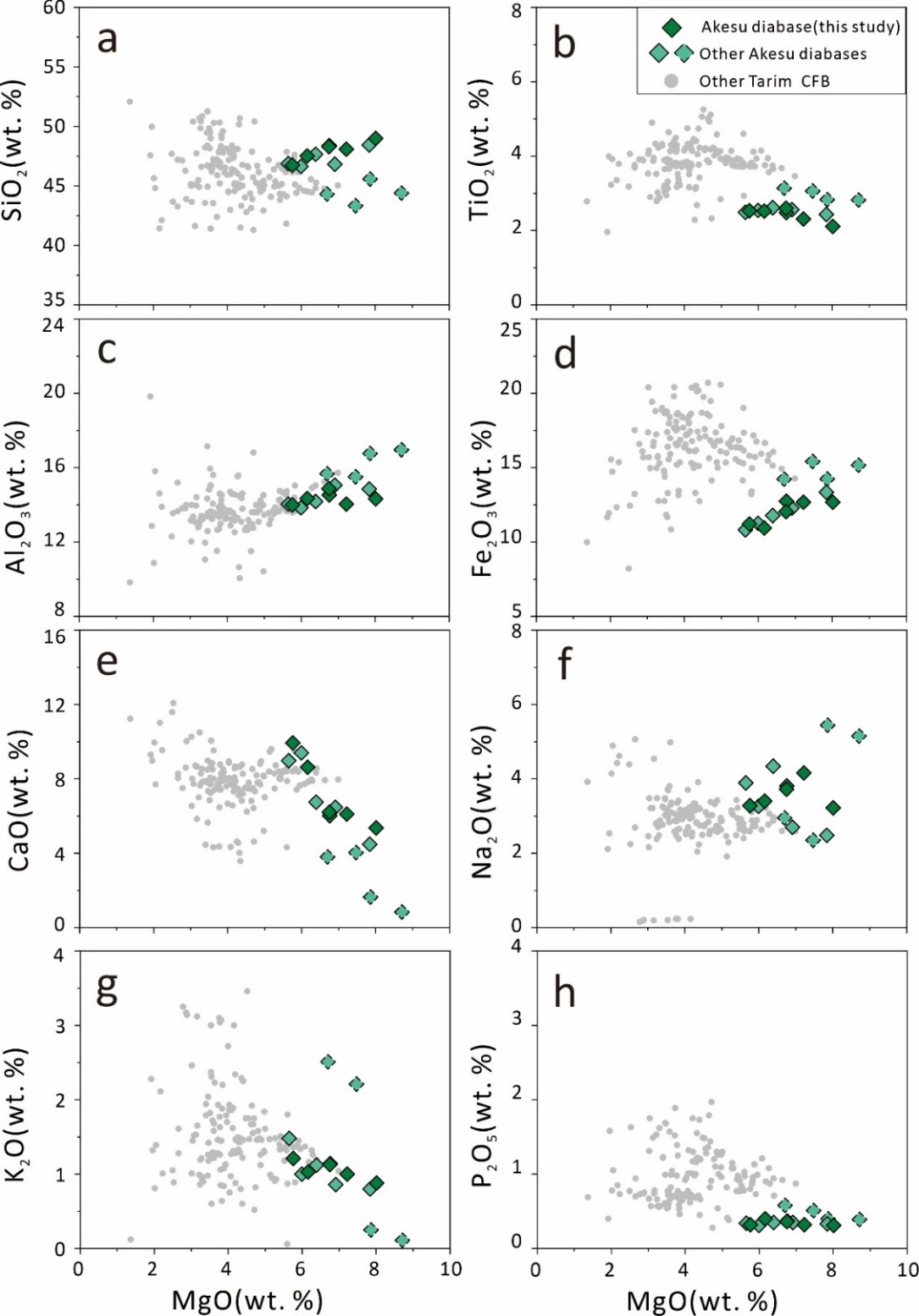


**Supplementary Figure S1.** Major elements oxide versus MgO for the Akesu diabase and other Tarim CFB. The data symbols with dashed borders represent samples that have been strongly affected by alteration (Supplementary Fig. S7). The data of the Akesu diabase are from Cheng *et al*.^15^. The data of other Tarim CFB are from the Sishichang section, the Yigan section, the Xiahenan section, the Damusi section and the Qipan section, respectively^4-9,11,21-27^.


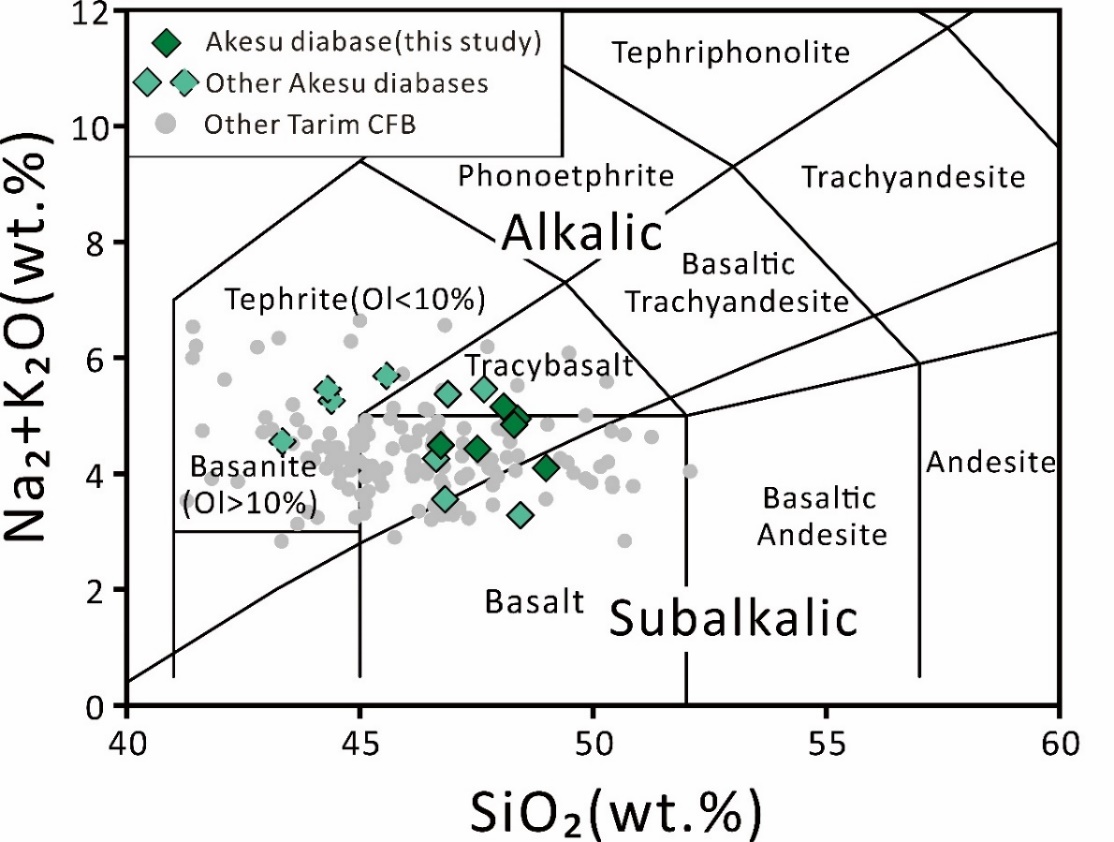


**Supplementary Figure S2.** The TAS diagram for the Akesu diabase and other Tarim CFB (modified after Le Bas and others^28^). The Alkalic-Subalkalic divide is from Irvine and Baragar^29^. The data source of the Akesu diabase and other Tarim CFB is same as in Supplementary Fig. S1.


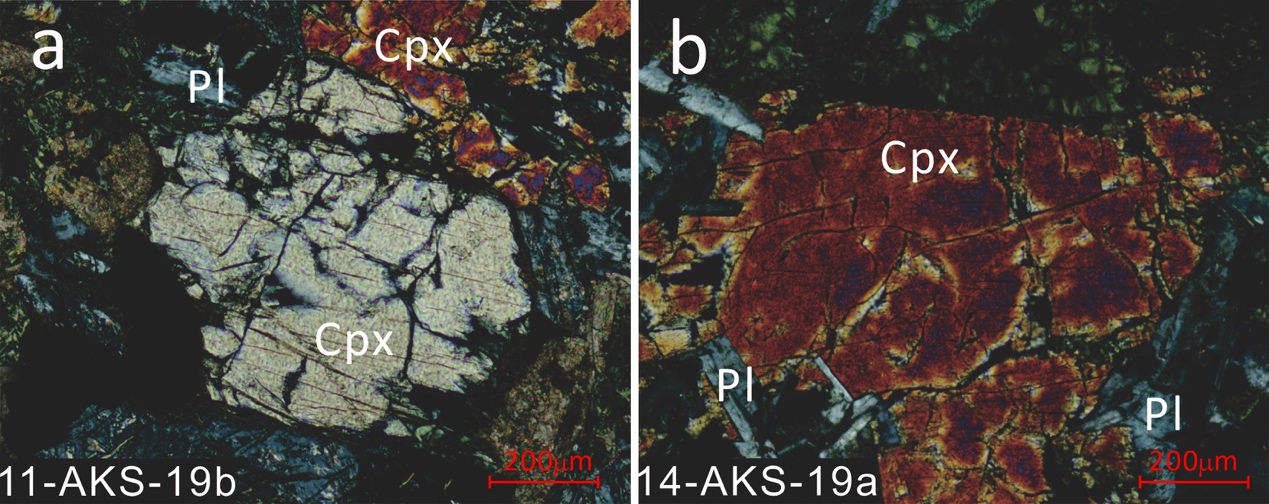


**Supplementary Figure S3.** Petrographic microscope images of clinopyroxene phenocrysts from the Akesu diabase. Abbreviations: Cpx= Clinopyroxene Pl=Plagioclase.

**
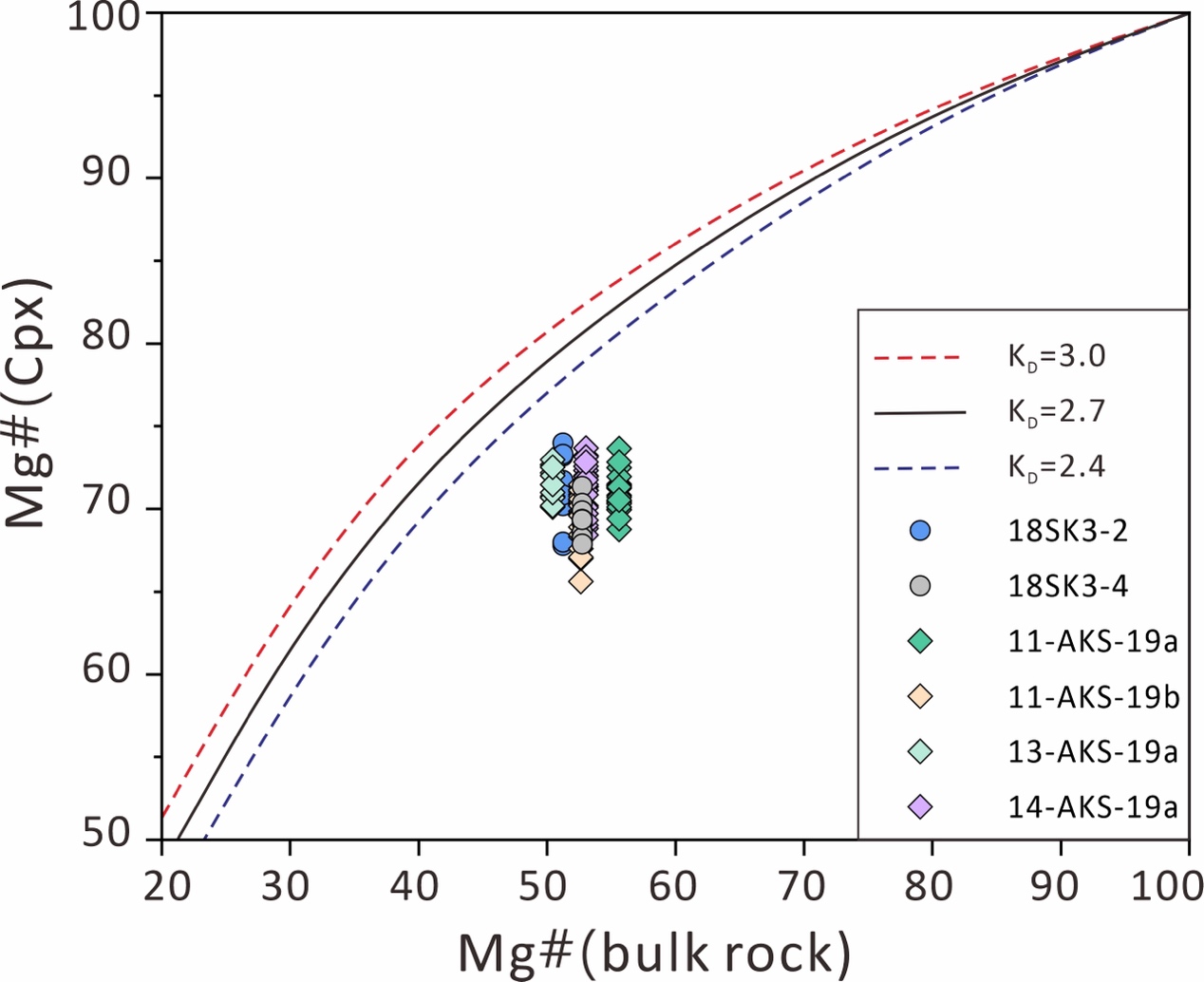
**

**Supplementary Figure S4.** Rhodes diagram for the clinopyroxene in the Akesu diabase. K_D_^(Fe-Mg)^_cpx-liq_=2.7±0.3^30^. The Mg# value of bulk rock is calculated based on the data from Akesu diabase, sourced from Cheng *et al.*^15^. Abbreviations: Cpx= Clinopyroxene.

**
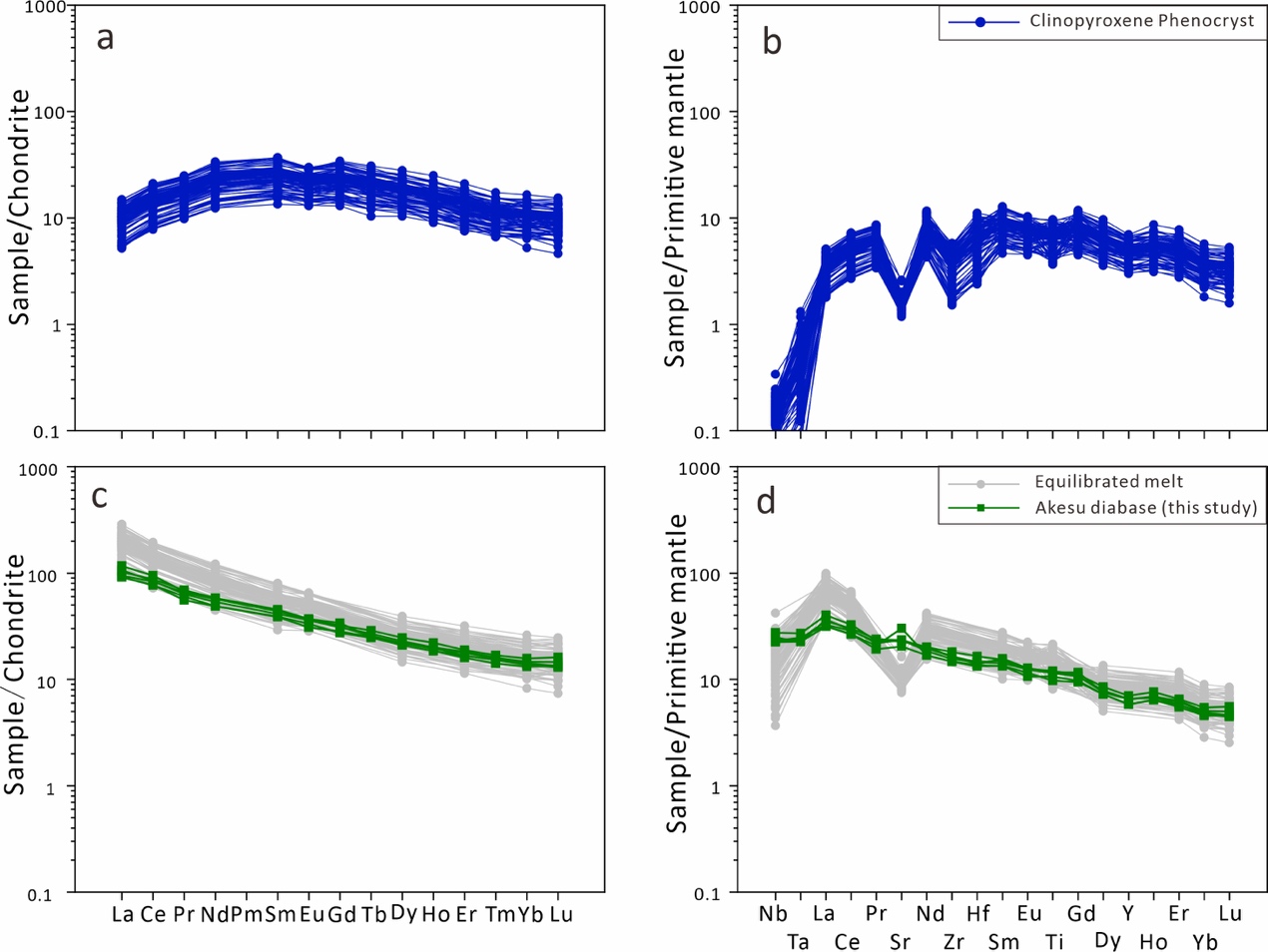
**

**Supplementary Figure S5.** (a) Chondrite-normalized REE diagram and (b) Primitive mantle-normalized variation diagram for the clinopyroxene phenocryst in the Akesu diabase. (c) Calculated Chondrite-normalized REE diagram and (d) Primitive mantle-normalized variation diagram for the equilibrated melt and the Akesu diabase. The partition coefficients for trace elements between the clinopyroxene and equilibrated melt are from Hauri *et al*^31^. Normalizing values are from Sun and McDonough^32^. The data of the Akesu diabase are from Cheng *et al.*^15^.


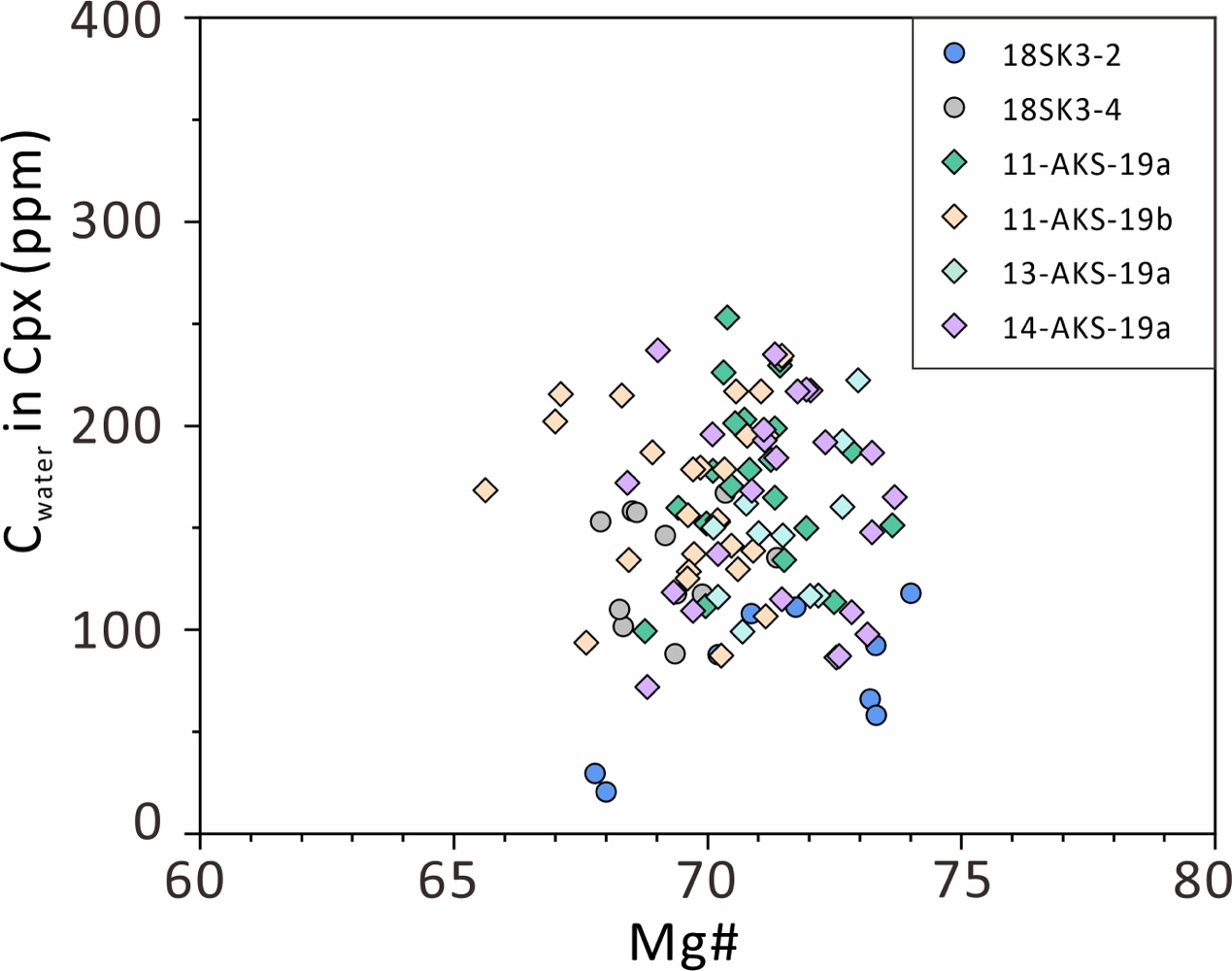


**Supplementary Figure S6.** Water contents versus Mg# values of clinopyroxene phenocrysts in the Akesu diabase. Abbreviations: Cpx= Clinopyroxene.


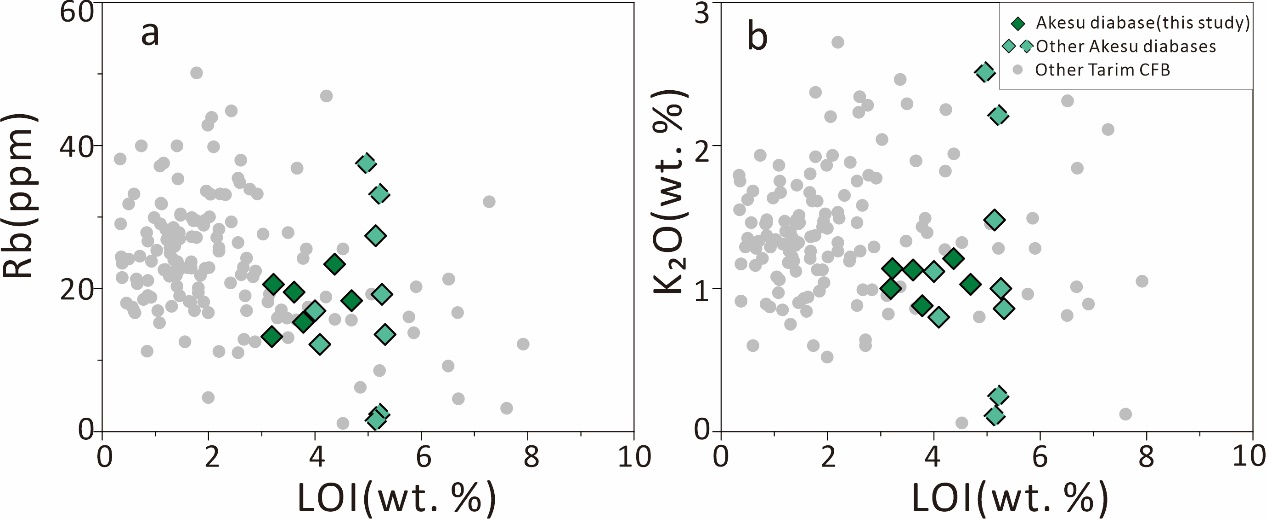


**Supplementary Figure S7.** (a) Rb and (b) K_2_O versus LOI diagram for the Akesu diabase and other Tarim CFB. Abbreviations: LOI=loss on ignition. The data source of Akesu diabase and other Tarim CFB is same as in Supplementary Fig. S1.

**
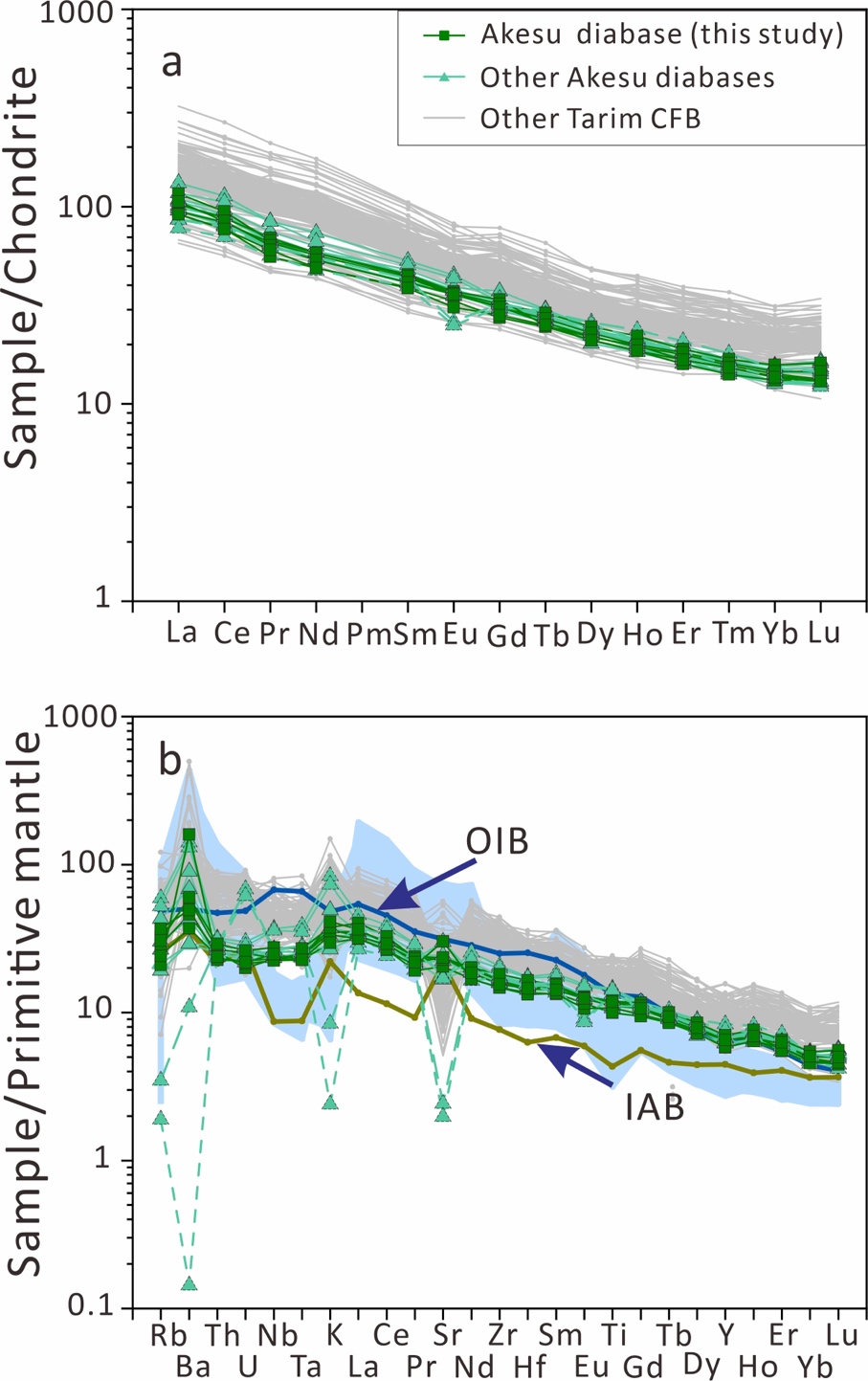
**

**Supplementary Figure S8.** (a) Chondrite-normalized REE diagram and (b) Primitive mantle-normalized variation diagram for the Akesu diabase and other Tarim CFB. The blue area delineates the range of the Mesozoic continental basalt from North China Craton. Normalizing values and the data of ocean island basalt are from Sun and McDonough^32^. The data of Mesozoic continental basalt from North China Craton are from Feixian^19^, Yixian^33^ and Sihetun^33^, respectively. The data of island arc basalt are from Ryukyu^34^. The data source of Akesu diabase and other Tarim CFB is same as in Supplementary Fig. S1.

**
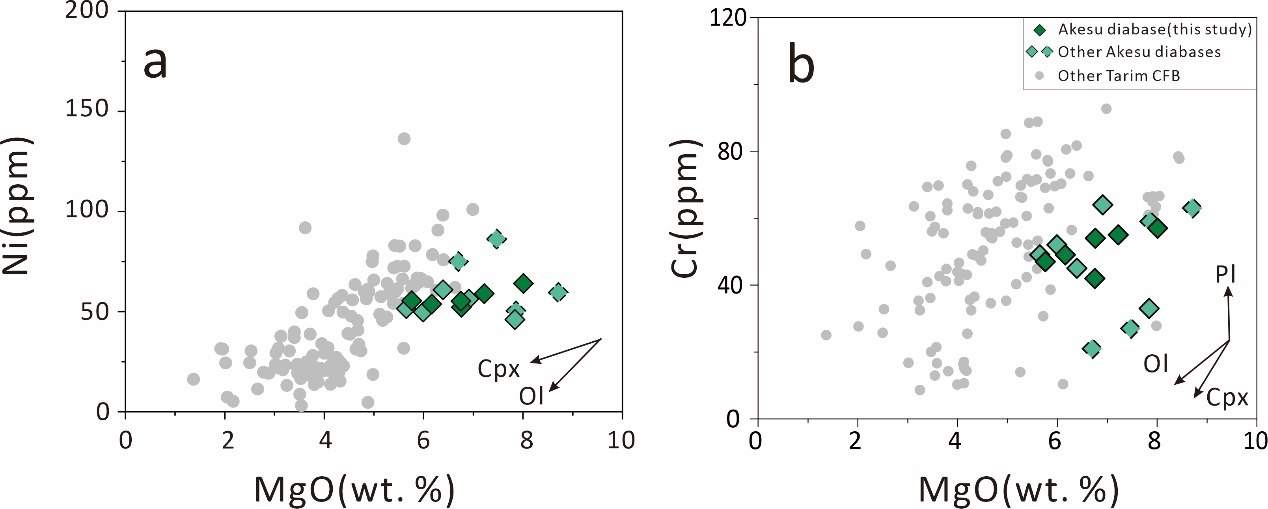
**

**Supplementary Figure S9.** (a) Ni and (b) CaO/Al_2_O_3_ versus MgO diagram for Akesu diabase and other Tarim CFB. The arrows indicate the effect of crystallization of different minerals. Abbreviations: Ol = Olivine, Cpx = Clinopyroxene, Pl = Plagioclase. The data source of Akesu diabase and other Tarim CFB is same as in Fig. S1.


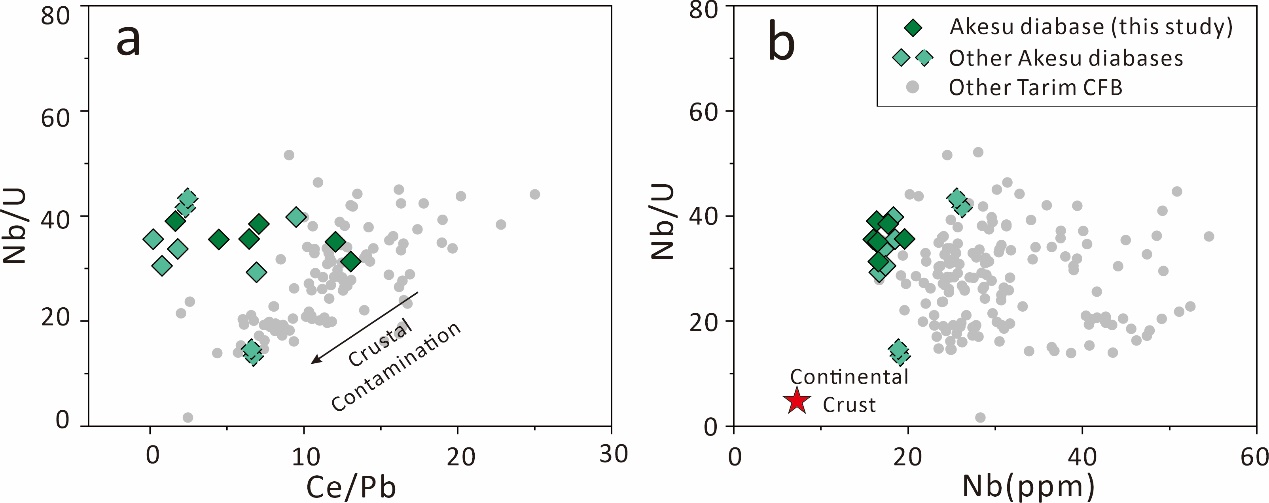


**Supplementary Figure S10.** (a) Nb/U versus Ce/Pb diagram and (b) Nb/U versus Nb diagram for the Akesu diabase and other Tarim CFB. The elemental composition of the continental crust is from Rudnick and Gao^35^. The data source of Akesu diabase and other Tarim CFB is same as in Supplementary Fig. S1.

**
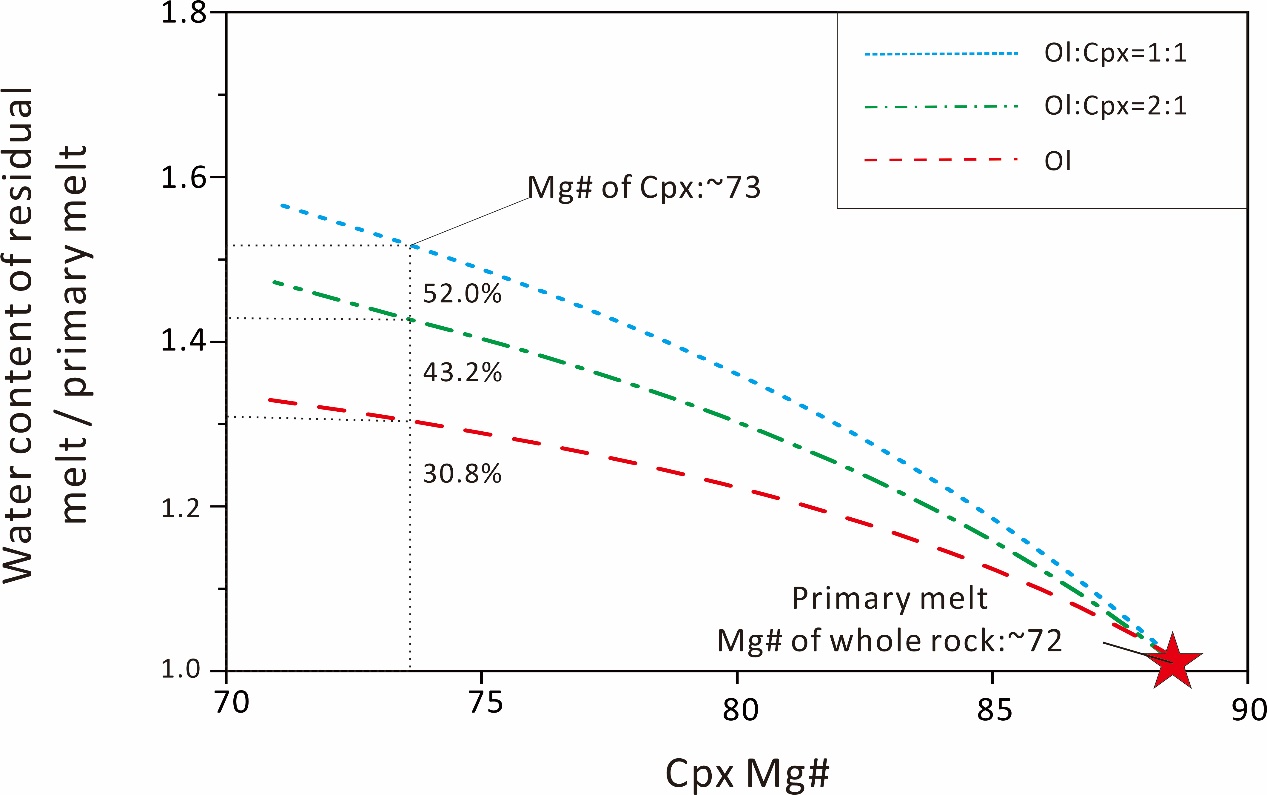
**

**Supplementary Figure S11** Evolution of water content in melt during the fractional crystallization of different mineral assemblages versus Mg# of clinopyroxene. The water partition coefficient between olivine and the melt is assumed to be 0.0002^36^; the Fe-Mg exchange coefficient (Kd) of olivine is 0.30^37^. The water partition coefficient between clinopyroxene and the melt is 0.015 (average value of clinopyroxene in the Akesu diabase, calculated using equation 10 in O'Leary et al., 2010)^38^; the Kd of clinopyroxene is 0.33^37^.


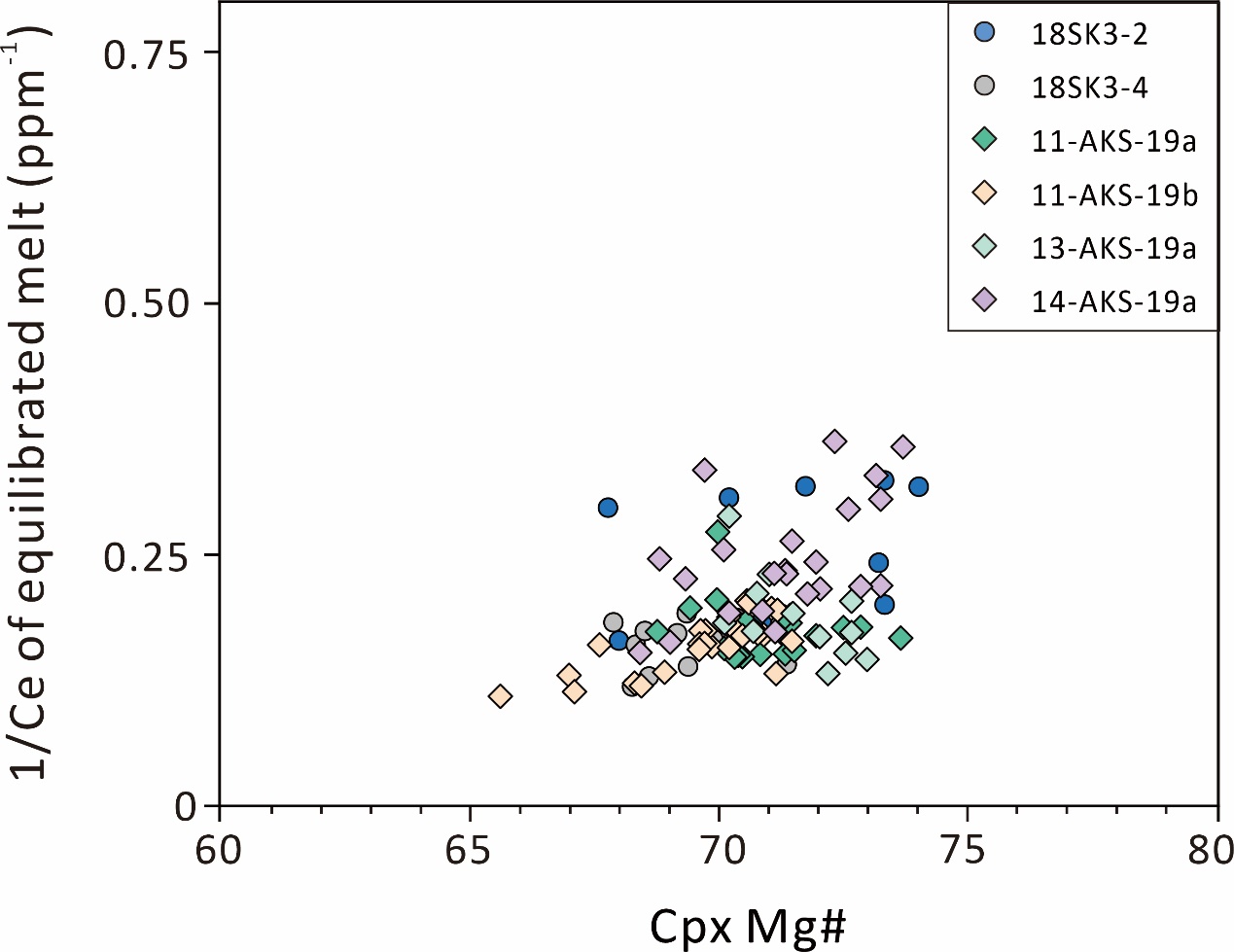


**Supplementary Figure S12** 1/Ce of equilibrated melt versus Mg# of clinopyroxene phenocrysts.


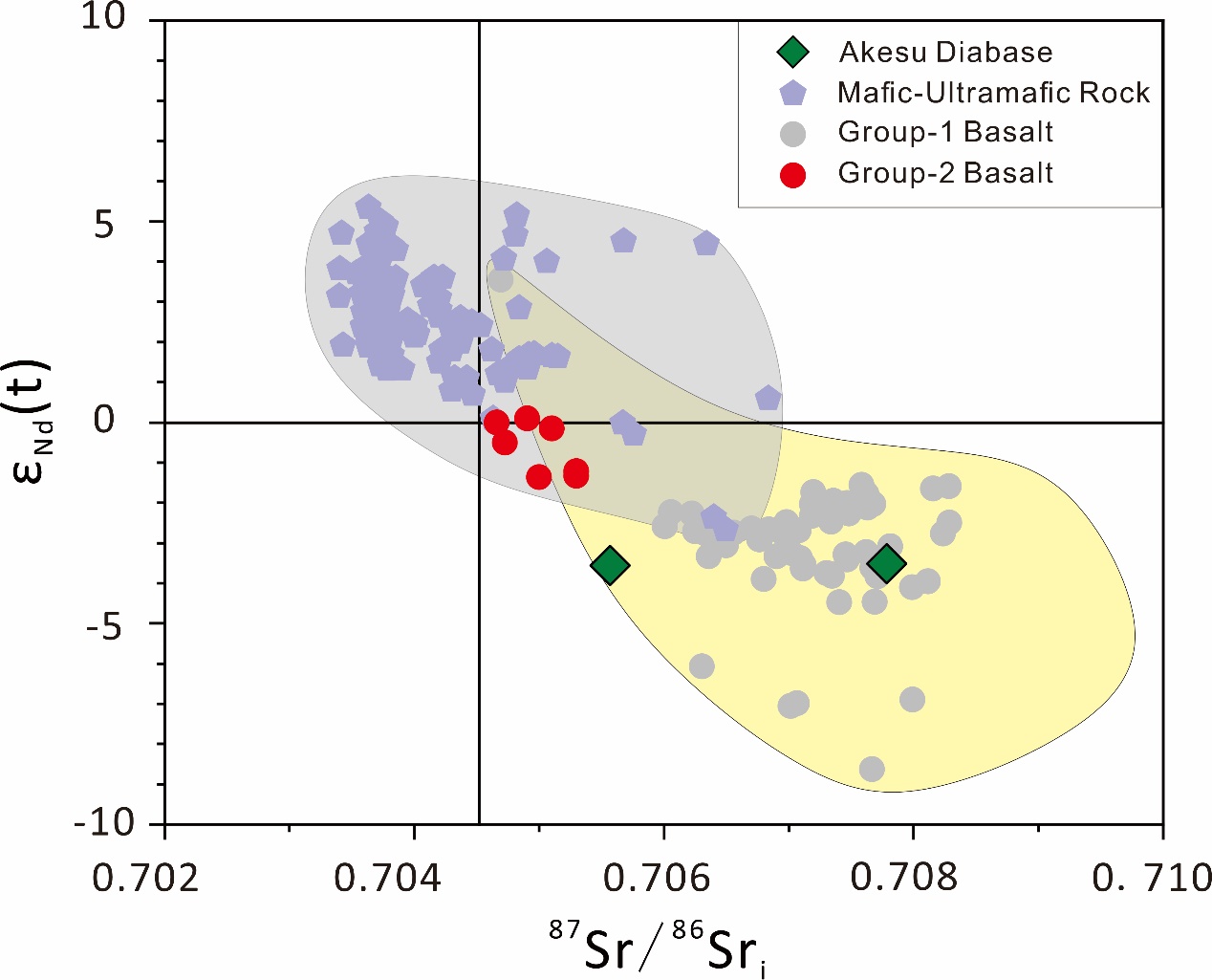


**Supplementary Figure S13** Initial Sr and Nd isotopic compositions of Akesu diabase and other Tarim LIP. The data source of Akesu diabase is from Cheng et al.^15^. The data source of Group-1 basalt is from Keping area^4,6-9,21-23^, while the data source of Group-2 basalt is from oil-drilling in the northern Tarim Basin^39^. The data source of ultramafic-mafic intrusion rocks is from Bachu and Wajilitage^6-9,22,23^ .

**References**

1 Yang, S. *et al*. Early Permian Tarim large igneous province in northwest China. *Science China Earth Sciences* **56,** 2015-2026; 10.1007/s11430-013-4653-y (2013).

2 Xu, Y. G., Wei, X., Luo, Z. Y., Liu, H. Q. & Cao, J. The Early Permian Tarim Large Igneous Province: main characteristics and a plume incubation model. *Lithos* **204,** 20-35; 10.1016/j.lithos.2014.02.015 (2014).

3 Cheng, Z. *et al.* Petrogenesis of Transitional Large Igneous Province: Insights From Bimodal Volcanic Suite in the Tarim Large Igneous Province. *Journal of Geophysical Research: Solid Earth* **125,** e2019JB018382; 10.1029/2019JB018382 (2020).

4 Yu, X. Magma evolution and deep geological processes of early Permian Tarim large igneous province. (Ph.D thesis) *Zhejiang University, Hangzhou* (2009).

5 Yu, X. *et al*. Permian flood basalts from the Tarim Basin, Northwest China: SHRIMP zircon U-Pb dating and geochemical characteristics. *Gondwana Research* **20,** 485-497; [10.1016/j.gr.2010.11.009](https://doi.org/10.1016/j.gr.2010.11.009) (2011).

6 Wei, X., Xu, Y. G., Feng, Y. X. & Zhao, J. X. Plume-lithosphere interaction in the generation of the Tarim large igneous province, NW China: Geochronological and geochemical constraints. *American Journal of Science* **314,** 314-356; 10.2475/01.2014.09 (2014).

7 Cheng, Z., Zhang, Z., Xie, Q., Hou, T. & Ke, S. Subducted slab-plume interaction traced by magnesium isotopes in the northern margin of the Tarim Large Igneous Province. *Earth and Planetary Science Letters* **489,** 100-110; [10.1016/j.epsl.2018.02.039](https://doi.org/10.1016/j.epsl.2018.02.039) (2018).

8 Wang, Z. *et al*. Tracing decarbonated eclogite in the mantle sources of Tarim continental flood basalts using Zn isotopes. *Geological Society of America Bulletin* B36502; 10.1130/B36502.1 (2022).

9 Zhou, M. F. *et al*. OIB-like, heterogeneous mantle sources of Permian basaltic magmatism in the western Tarim Basin, NW China: implications for a possible Permian large igneous province. *Lithos* **113,** 583-594; [10.1016/j.lithos.2009.06.027](https://doi.org/10.1016/j.lithos.2009.06.027) (2009).

10 Yang, S.-f., Li, Z.-l., Chen, H.-l., Chen, W. & Yu, X. ^40^Ar-^39^Ar dating of basalts from Tarim Basin, NW China and its implication to a Permian thermal tectonic event. [*Journal of Zhejiang University-science A*](https://www.infona.pl/resource/bwmeta1.element.springer-000000011582/tab/jContent) **7,** 320; 10.1631/jzus.2006.AS0320 (2006).

11 Li, H. *et al*. Age and geochemistry of the Early Permian basalts from Qimugan in the southwestern Tarim basin. *Acta Petrologica Sinica* **29**, 3353-3368; 1000-0569/2013/029(10)-3353-68 (2013).

12 Zhong, Y.-T. *et al.* Constraining the duration of the Tarim flood basalts (northwestern China): CA-TIMS zircon U-Pb dating of tuffs. *Geological Society of America Bulletin* **134,** 325-334; 10.1130/b36053.1 (2021)

13 Li, Z. *et al.* Temporal evolution of the Permian large igneous province in Tarim Basin in northwestern China. *Journal of Asian Earth Sciences* **42,** 917-927; [10.1016/j.jseaes.2011.05.009](https://doi.org/10.1016/j.jseaes.2011.05.009) (2011).

14 Wu, H. *et al.* Discovery of Permian mafic sills intrusion event in the Sinian System, Northwest Tarim block. *Acta Geologica Sinica* **94,** 1869-1882; 10.19762/j.cnki.dizhixuebao.2020079 (2020).

15 Cheng, X. X. *et al.* The Permian mafic intrusive events in the northwestern margin of the Tarim Basin and their tectonic significance. *Acta Petrologica Sinica* **38,** 743-764; 10.18654/1000-0569/2022.03.09 (2022).

16 Bell, D. R., Ihinger, P. D. & Rossman, G. R. Quantitative analysis of trace OH in garnet and pyroxenes. *American Mineralogist* **80,** 465-474; [10.2138/am-1995-5-607](https://doi.org/10.2138/am-1995-5-607) (1995).

17 Kovács, I. *et al*. Quantitative absorbance spectroscopy with unpolarized light: Part II. Experimental evaluation and development of a protocol for quantitative analysis of mineral IR spectra. *American Mineralogist* **93,** 765-778; [10.2138/am.2008.2656](https://doi.org/10.2138/am.2008.2656) (2008).

18 Sambridge, M., Gerald, J. F., Kovács, I., O’Neill, H. S. C. & Hermann, J. R. Quantitative absorbance spectroscopy with unpolarized light: Part I. Physical and mathematical development. *American Mineralogist* **93,** 751-764; [10.2138/am.2008.2656](https://doi.org/10.2138/am.2008.2656) (2008).

19 Xia, Q. K. *et al*. High water content in Mesozoic primitive basalts of the North China Craton and implications on the destruction of cratonic mantle lithosphere. *Earth and Planetary Science Letters* **361,** 85-97; [10.1016/j.epsl.2012.11.024](https://doi.org/10.1016/j.epsl.2012.11.024) (2013).

20 Liu, J. *et al.* Water Content and Oxygen Isotopic Composition of Alkali Basalts from the Taihang Mountains, China: Recycled Oceanic Components in the Mantle Source. *Journal of Petrology* **56,** 681-702; 10.1093/petrology/egv013 (2015).

21 Jiang, C. *et al*. Petrology, geochemistry and petrogenesis of the Kalpin basalts and their Nd, Sr and Pb isotopic compositions. *Geological Review* **50,** 492-500; 10.3321/j.issn:0371-5736.2004.05.007 (2004).

22 Zhang, Y., Liu, J. & Guo, Z. Permian basaltic rocks in the Tarim basin, NW China: implications for plume-lithosphere interaction. *Gondwana Research* **18,** 596-610; [10.1016/j.gr.2010.03.006](https://doi.org/10.1016/j.gr.2010.03.006) (2010).

23 Li, Y. Q. *et al*. Platinum-group elements and geochemical characteristics of the Permian continental flood basalts in the Tarim Basin, northwest China: implications for the evolution of the Tarim Large Igneous Province. *Chemical Geology* **328,** 278-289; [10.1016/j.chemgeo.2012.03.007](https://doi.org/10.1016/j.chemgeo.2012.03.007) (2012).

24 Yuan, F. *et al*. Siderophile and chalcophile metal variations in basalts: Implications for the sulfide saturation history and Ni-Cu-PGE mineralization potential of the Tarim continental flood basalt province, Xinjiang Province, China. *Ore Geology Reviews* **45,** 5-15; [10.1016/j.oregeorev.2011.04.003](https://doi.org/10.1016/j.oregeorev.2011.04.003) (2012).

25 Dai, Y. *et al*. Zircon U-Pb chronology, geochemical characteristics of the Early Permian basalt in the Keping area, Xinjiang and their geological significance. *Geological Science and Technology Information* **36,** 1-13; 10.19509/j.cnki.dzkq.2017.0101 (2017).

26 Yu, X. *et al*. Petrogeochemical characteristics and geological implications of layered basalts from Xiahenan area, Tarim Basin. *Acta Petrologica Sinica* **33,** 1729-1740; 1000-0569/2017/033(06)-1729 (2017).

27 Li, Z. *et al*. Chronology and geochemistry of Taxinan basalts from the Tarim basin: evidence for Permian plume magmatism. *Acta Petrologica Sinica* **24,** 959-970; 1000-0569/2008/024(05)-0959-70 (2008).

28 Le Bas, M. L., Le Maitre, R. W., Streckeisen, A. & Zanettin, B. A chemical classification of volcanic rocks based on the total alkali-silica diagram. *Journal of petrology* **27,** 745-750; [10.1093/petrology/27.3.745](https://doi.org/10.1093/petrology/27.3.745) (1986).

29 Irvine, T. N. & Baragar, W. A guide to the chemical classification of the common volcanic rocks. *Canadian journal of earth sciences* **8**, 523-548; [10.1139/e71-055](https://doi.org/10.1139/e71-055) (1971).

30 Villiger, S., Ulmer, P. & Müntener, O. Equilibrium and fractional crystallization experiments at 0· 7 GPa; the effect of pressure on phase relations and liquid compositions of tholeiitic magmas. *Journal of Petrology* **48,** 159-184; [10.1093/petrology/egl058](https://doi.org/10.1093/petrology/egl058) (2007).

31 Hauri, E. H., Wagner, T. P. & Grove, T. L. Experimental and natural partitioning of Th, U, Pb and other trace elements between garnet, clinopyroxene and basaltic melts. *Chemical Geology* **117**, 149-166 10.1016/0009-2541(94)90126-0 (1994).

32 Sun, S. S. & McDonough, W. F. Chemical and isotopic systematics of oceanic basalts: implications for mantle composition and processes. *Geological Society, London, Special Publications* **42**, 313-345; [10.1144/GSL.SP.1989.042.01.19](https://doi.org/10.1144/GSL.SP.1989.042.01.19) (1989).

33 Geng, X. *et.al.* Thermal-chemical conditions of the North China Mesozoic lithospheric mantle and implication for the lithospheric thinning of cratons. *Earth and Planetary Science Letters*, **516,** 1-11; 10.1016/j.epsl.2019.03.012 (2019).

34 Chen, Z. *et al.* Zinc isotopes of the Mariana and Ryukyu arc‐related lavas reveal recycling of forearc serpentinites into the subarc mantle. *Journal of Geophysical Research: Solid Earth* **126,** e2021JB022261; [10.1029/2021JB022261](https://doi.org/10.1029/2021JB022261) (2021).

35 Rudnick, R., Gao, S., Holland, H. & Turekian, K. Composition of the continental crust in *The crust* (eds. Rudnick, R. L.) 1-64 (Elsevier, 2003).

36 Hauri, E. H., Gaetani, G. A. & Green, T. H. Partitioning of water during melting of the Earth's upper mantle at H_2_O-undersaturated conditions. *Earth and Planetary Science Letters* **248,** 715-734; [10.1016/j.epsl.2006.06.014](https://doi.org/10.1016/j.epsl.2006.06.014) (2006).

37 Roeder, P. & Emslie, R. Olivine-liquid equilibrium. *Contributions to mineralogy and petrology* **29,** 275-289; 10.1007/BF00371276 (1970).

38 O'Leary, J. A., Gaetani, G. A. & Hauri, E. H. The effect of tetrahedral Al^3+^ on the partitioning of water between clinopyroxene and silicate melt. *Earth and Planetary Science Letters* **297,** 111-120; [10.1016/j.epsl.2010.06.011](https://doi.org/10.1016/j.epsl.2010.06.011) (2010).

39 Tian, W. et al. The Tarim picrite–basalt–rhyolite suite, a Permian flood basalt from northwest China with contrasting rhyolites produced by fractional crystallization and anatexis. *Contributions to Mineralogy and Petrology* **160**, 407–425; 10.1007/s00410-009-0485-3 (2010).
